# Synthesis, Optimization, Antifungal Activity, Selectivity, and CYP51 Binding of New 2-Aryl-3-azolyl-1-indolyl-propan-2-ols

**DOI:** 10.3390/ph13080186

**Published:** 2020-08-08

**Authors:** Nicolas Lebouvier, Fabrice Pagniez, Young Min Na, Da Shi, Patricia Pinson, Mathieu Marchivie, Jean Guillon, Tarek Hakki, Rita Bernhardt, Sook Wah Yee, Claire Simons, Marie-Pierre Lézé, Rolf W. Hartmann, Angélique Mularoni, Guillaume Le Baut, Isabelle Krimm, Ruben Abagyan, Patrice Le Pape, Marc Le Borgne

**Affiliations:** 1 EA1155—IICiMed, Institut de Recherche en Santé 2, Département de Pharmacochimie, Nantes Atlantique Universités, Université de Nantes, F-44200 Nantes, France; nicolas.lebouvier@univ-nc.nc (N.L.); ymna@joowonpat.com (Y.M.N.); patricia.pinson@univ-nantes.fr (P.P.); mpl_leze@yahoo.fr (M.-P.L.); guillaume.lebaut@wanadoo.fr (G.L.B.); 2 Institut des Sciences Exactes et Appliquées (ISEA) EA 7484, Université de la Nouvelle-Calédonie, 98851 Noumea CEDEX, New Caledonia; 3 EA1155—IICiMed, Institut de Recherche en Santé 2, Département de Parasitologie et Mycologie Médicale, Nantes Atlantique Universités, Université de Nantes, F-44200 Nantes, France; fabrice.pagniez@univ-nantes.fr; 4 Skaggs School of Pharmacy and Pharmaceutical Sciences, University of California, San Diego, CA 92093, USA; das046@ucsd.edu (D.S.); ruben@ucsd.edu (R.A.); 5 ICMCB CNRS-UPR 9048, Université de Bordeaux, F-33608 Pessac CEDEX, France; mathieu.marchivie@icmcb.cnrs.fr; 6 ARNA Laboratory, INSERM U1212, UMR CNRS 5320, UFR des Sciences Pharmaceutiques, Université de Bordeaux, F-33076 Bordeaux, France; jean.guillon@u-bordeaux.fr; 7 Department of Biochemistry, Saarland University, Campus B2.2, 66123 Saarbrücken, Germany; t.hakki@dr-hakki.de (T.H.); ritabern@mx.uni-saarland.de (R.B.); 8 School of Pharmacy & Pharmaceutical Sciences, Cardiff University, King Edward VII Avenue, Cardiff CF10 3NB, UK; sookwah.yee@ucsf.edu (S.W.Y.); SimonsC@cardiff.ac.uk (C.S.); 9 Department of Pharmacy, Pharmaceutical and Medicinal Chemistry, Saarland University, Campus E8.1, 66123 Saarbrücken, Germany; Rolf.Hartmann@helmholtz-hzi.de; 10 EA 4446 Bioactive Molecules and Medicinal Chemistry, Faculté de Pharmacie-ISPB, SFR Santé Lyon-Est CNRS UMS3453-INSERM US7, Université Claude Bernard Lyon 1, Université de Lyon, CEDEX 8, F-69373 Lyon, France; angelique.mularoni@univ-lyon1.fr; 11 Centre de RMN à Très Hauts Champs, CNRS, ENS, Université Claude Bernard Lyon 1, Université de Lyon, 5 rue de la Doua, F-69100 Villeurbanne, France; isabelle.krimm@univ-lyon1.fr; 12 Centre de Recherche en Cancérologie de Lyon, INSERM 1052, CNRS 5286, Centre Léon Bérard, Université Claude Bernard Lyon 1, Université de Lyon, F-69008 Lyon, France

**Keywords:** azoles, antifungal agents, indole, microwave irradiation, X-ray crystallography, *Candida* species, docking, CYP51, selectivity, cytochromes P450

## Abstract

A series of 2-aryl-3-azolyl-1-indolyl-propan-2-ols was designed as new analogs of fluconazole (FLC) by replacing one of its two triazole moieties by an indole scaffold. Two different chemical approaches were then developed. The first one, in seven steps, involved the synthesis of the key intermediate 1-(1*H*-benzotriazol-1-yl)methyl-1*H*-indole and the final opening of oxiranes by imidazole or 1*H*-1,2,4-triazole. The second route allowed access to the target compounds in only three steps, this time with the ring opening by indole and analogs. Twenty azole derivatives were tested against *Candida albicans* and other *Candida* species. The enantiomers of the best anti-*Candida* compound, 2-(2,4-dichlorophenyl)-3-(1*H*-indol-1-yl)-1-(1*H*-1,2,4-triazol-1-yl)-propan-2-ol (**8g**), were analyzed by X-ray diffraction to determine their absolute configuration. The (−)-**8g** enantiomer (Minimum inhibitory concentration (MIC) = IC_80_ = 0.000256 µg/mL on *C. albicans* CA98001) was found with the *S*-absolute configuration. In contrast the (*+*)-**8g** enantiomer was found with the *R*-absolute configuration (MIC = 0.023 µg/mL on *C. albicans* CA98001). By comparison, the MIC value for FLC was determined as 0.020 µg/mL for the same clinical isolate. Additionally, molecular docking calculations and molecular dynamics simulations were carried out using a crystal structure of *Candida albicans* lanosterol 14α-demethylase (CaCYP51). The (−)-(*S*)-**8g** enantiomer aligned with the positioning of posaconazole within both the heme and access channel binding sites, which was consistent with its biological results. All target compounds have been also studied against human fetal lung fibroblast (MRC-5) cells. Finally, the selectivity of four compounds on a panel of human P450-dependent enzymes (CYP19, CYP17, CYP26A1, CYP11B1, and CYP11B2) was investigated.

## 1. Introduction

Invasive fungal infections are related to a high mortality rate despite the availability of several antifungal drugs. Fungi species that belong to one of these four genera (*Cryptococcus*, *Candida*, *Aspergillus*, and *Pneumocystis*) kill about one and a half million people every year [1,2]. These infections have increased in frequency and severity over the last two decades as a result of an increasing number of immunocompromised hosts due to cancer chemotherapy, organ and bone marrow transplantation, human immunodeficiency virus, or therapy against autoimmune and inflammatory diseases especially with TNF inhibitors [3]. *Candida albicans* (*C. albicans*) is the major opportunistic pathogen of fungal infections and *Candida* spp. are the fourth most common nosocomial bloodstream pathogens in the USA with a mortality rate of 40% [1,2].

For many years, the polyene amphotericin B has been the only available antifungal drug for invasive fungal infections despite nephrotoxicity, infusion-related reactions, and other adverse effects [4]. To this day, approved antifungal agents belong to only four drug classes: Polyenes, azoles, echinocandins, and anti-metabolites [5]. Introduction of triazoles (e.g., fluconazole (FLC), itraconazole) offered new treatment options with excellent antifungal activity and a good safety profile. Prophylactic use of FLC has been developed to reduce the risk of *Candida* colonization and infection in high-risk patients with minimal toxicity [6,7]. Nevertheless, the emergence of *C. krusei* and *C. glabrata* infections in patients with bone marrow transplantation or neutropenia receiving FLC prophylaxis has been reported [8,9]. Development of *Candida* spp. resistance has also commonly been observed in HIV-infected patients who received prolonged FLC treatment for oropharyngeal candidiasis [2,10,11].

Fourth generation azoles (e.g., voriconazole, posaconazole, ravuconazole, isavuconazole) and echinocandins (e.g., caspofungin, micafungin, anidulafungin) approved for use or under clinical trials have proven to be less toxic than conventional amphotericin B, resulting in a significant advance in treatment of serious fungal infections. However, voriconazole interferes with many drugs and its prolonged use may expose severely immunocompromised patients to the risk of zygomycosis [12], while echinocandins are not available orally [13]. Consequently, most patients requiring therapy for invasive candidiasis are still placed on the safe, inexpensive, and orally available fluconazole. Despite recent advances in antifungal chemotherapy, the mortality rates of invasive fungal infections have remained unchanged and there is a necessity for the development of new antifungal compounds. Aiming at new and effective antifungal drugs, our attention was focused on the azole derivatives because of their low toxicity, broad spectrum of activity, and favorable pharmacokinetic properties [10,11,14].

**Figure 1:**
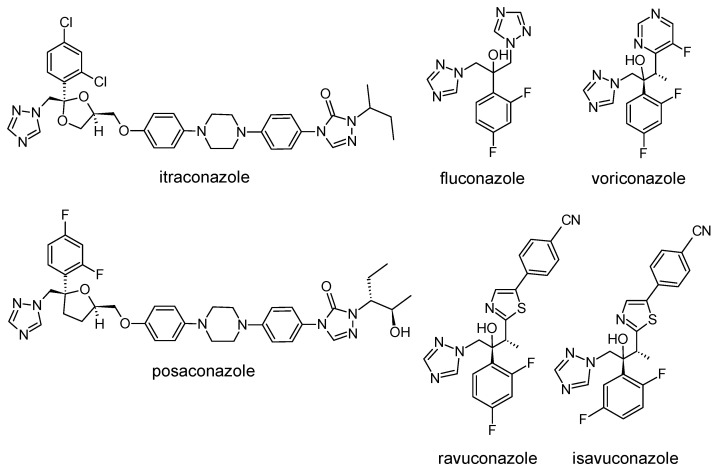
Structures of the main azole antifungal agents.

Antifungal azoles (Figure 1) target the P450-dependent enzyme lanosterol 14α-demethylase (CYP51), preventing the production of ergosterol, a key component in the fungal cell membrane [15]. Depletion of ergosterol and accumulation of 14α-methylated sterols alter membrane fluidity, increase permeability, and reduce the activity of membrane-associated enzymes. Azoles bind to the iron of the protoporphyrin unit located in the active site of CYP51, preventing the access of the natural substrate lanosterol [16,17]. Azoles are also used for the treatment of estrogen-dependent disease by inhibition of aromatase (CYP19) in breast cancer therapy [18,19], and the inhibition of human P450-dependent enzymes is an unwanted side effect that could lead to impediments for safe therapeutic profile, including toxicity and undesirable drug interactions [20]. Selectivity of antifungal agents is therefore crucial because of the high levels of similarity between the eukaryotic fungal pathogens and the human hosts, and must be considered as a top priority in the development of new antifungal azoles [5,10,21].

Our research group has developed several potent series of antifungal compounds based on an indole scaffold with better in vitro antifungal activities against *C. albicans* compared with FLC [22,23,24,25,26,27]. To continue this pharmacomodulation strategy, we describe in the present paper the synthesis of 2-phenyl-1-(1*H*-indol-1-yl)-3-(azolyl)propan-2-ols using two different synthetic routes and their anti-*Candida* activities. The safety profile of target compounds was also evaluated by a cytotoxicity assay on human fetal lung fibroblast (MRC-5) cells, and by selectivity assessment using a panel of human P450-dependent enzymes. Additionally, separation of the two enantiomers of the most potent compound, 2-(2,4-dichlorophenyl)-3-(1*H*-indol-1-yl)-1-(1*H*-1,2,4-triazol-1-yl)-propan-2-ol **8g**, was performed and the determination of their absolute configuration was achieved. A molecular modeling study was finally performed to assess the binding feasibility of (*R*)-**8g** and (*S*)-**8g** for lanosterol 14α-demethylase (CYP51).

## 2. Results and Discussion

### 2.1. Chemistry

Two synthetic routes were designed to obtain the 2-phenyl-3-(1*H*-indol-1-yl)-1-(azol-1-yl)propan-2-ol derivatives **8a**–**g** and **11a**–**l**.

**Scheme 1:**
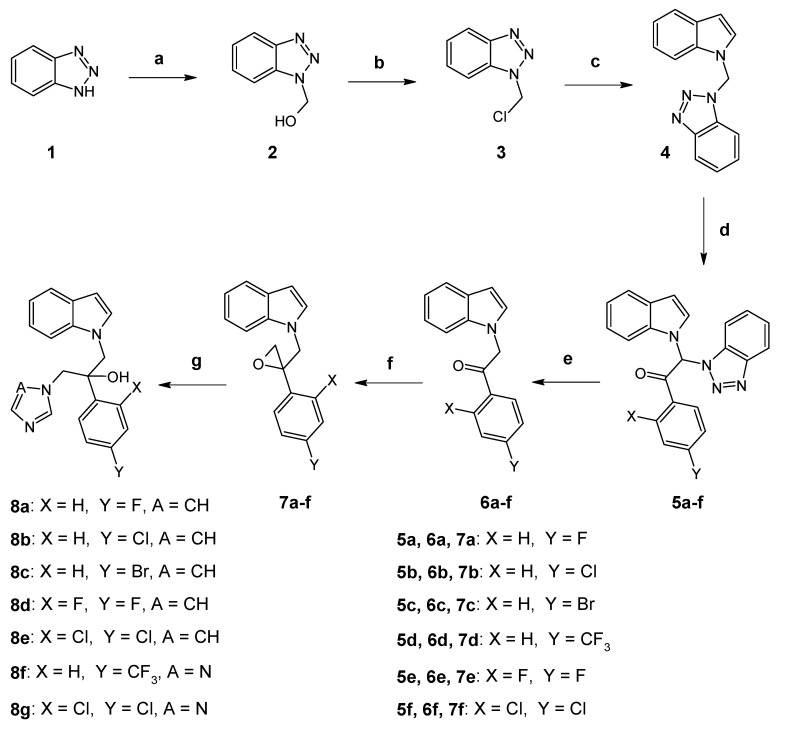
Synthesis of compounds **8a**–**g** (Route 1). Reagents and conditions: (**a**) HCHO_aq_, room temperature (rt), 5 min; (**b**) SOCl_2_, reflux, 1 h; (**c**) NaH, 1*H*-indole, DMSO, rt, 12 h; (**d**) *n*-BuLi, ethylbenzoate derivatives, THF, −78 °C to rt, 12 h; (**e**) AcOH, Zn, THF, EtOH, ultrasound, 35 °C, 5 h; (**f**) TMSOI, NaOH_aq_, CH_2_Cl_2_, reflux, 48 h; (**g**) K_2_CO_3_, imidazole or 1*H*-1,2,4-triazole, CH_3_CN, reflux, 7 h.

The first pathway strategy (route 1) involved the synthesis of the key intermediate 1-(1*H*-benzotriazol-1-yl)methyl-1*H*-indole **4** (Scheme 1). According to this scheme, hydroxymethylation of benzotriazole **1** using an aqueous solution of formaldehyde quickly gave 1-hydroxymethyl-1*H*-benzotriazole **2**, which was converted into 1-chloromethyl-1*H*-benzotriazole **3** using thionyl chloride at 0 °C. The indole was then alkylated by compound **3** in the presence of sodium hydride (NaH) in dimethyl sulfoxide (DMSO) to give the intermediate **4**. Lithiation of **4** with *n*-butyllithium at −78 °C in tetrahydrofuran (THF) followed by addition of the appropriate esters gave the target α-benzotriazolyl ketones **5a**–**f**. Debenzotriazolylation of **5a**–**f** occurred in the presence of zinc and acetic acid in a mixture of THF/EtOH (1:1, *v*/*v*) under ultrasonic activation. The corresponding ketones **6a**–**f** were then converted into oxiranes **7a**–**f** by the Corey–Chaykovsky epoxidation using trimethylsulfoxonium iodide (TMSOI) and an aqueous solution of sodium hydroxide in dichloromethane under reflux. The ring opening of **7a**–**f** with 1*H*-1,2,4-triazole or imidazole in the presence of potassium carbonate (K_2_CO_3_) at reflux in acetonitrile (CH_3_CN) gave the target 2-aryl-3-(1*H*-indol-1-yl)-1-(azol-1-yl)propan-2-ol derivatives **8a**–**g**.

**Scheme 2:**
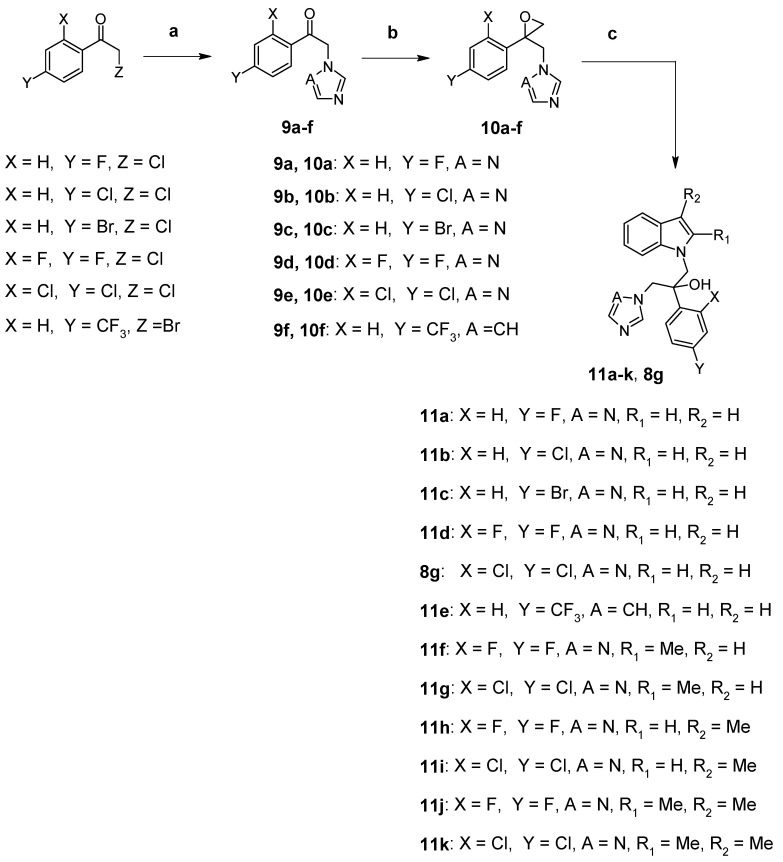
Synthesis of compounds **8g** and **11a**–**k** (Route 2). Reagents and conditions: (**a**) K_2_CO_3_, imidazole or 1*H*-1,2,4-triazole, CH_3_CN, MW 85 °C, 50 W, 50 min; (**b**) NaOH_aq_ 20%, TMSOI, CH_2_Cl_2_, reflux, 72 h; (**c**) NaH, indole derivative, DMSO, rt, 12 h.

In order to reduce the number of reaction steps, we investigated another synthetic pathway called route 2 (Scheme 2). First, imidazole and 1*H*-1,2,4-triazole were alkylated by commercially available halogenoacetophenones using K_2_CO_3_ in CH_3_CN under microwave heating (85 °C, 50 W) to give the target ketones **9a**–**f**, which were converted to oxiranes **10a**–**f** in the presence of TMSOI and an aqueous solution of sodium hydroxide in toluene under microwave heating (80 °C, 50 W). The reaction times of these two steps (alkylation and epoxidation) are greatly reduced by using microwave heating as we have already reported [27]. Finally, the ring opening of **10a**–**f** with indole using NaH in DMSO gave the derivatives **8g** and **11a**–**e**. A similar ring opening of the intermediates **9d** and **9e** by indoles with methyl groups in position 2 and/or 3 furnished the target derivatives **11f**–**k**. In the case of the synthesis of compound **8g**, route 2 gave a great improvement of the overall yield (route 1: 5% vs. route 2: 29%).

**Scheme 3:**
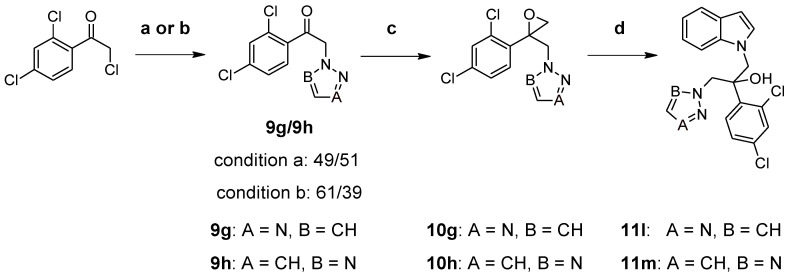
Synthesis of compounds **11l** and **11m**. Reagents and conditions: (**a**) K_2_CO_3_, 1*H*-1,2,3-triazole, CH_3_CN, MW 85 °C, 50 W, 50 min; (**b**) K_2_CO_3_, 1*H*-1,2,3-triazole, CH_3_CN, 85 °C, 8 h; (**c**) NaOH_aq_ 20%, TMSOI, CH_2_Cl_2_, reflux, 72 h; (**d**) NaH, indole, DMSO, rt, 12 h.

To observe the influence of the nitrogen atom position in the azole ring, the effective route 2 was used to synthesize two other 2-(2,4-dichlorophenyl)-3-(1*H*-indol-1-yl)-1-(azol-1-yl)propan-2-ol derivatives **11l** and **11m** with azole rings 1*H*-1,2,3-triazole and 2*H*-1,2,3-triazole (Scheme 3). Alkylation of 1*H*-1,2,3-triazole by 2,2′,4′-trichloroacetophenone gave a mixture of *N*-1 **9g** and *N*-2 **9h** alkylation products. This alkylation by conventional heating provided a ratio of 61/39 *N*-1 and *N*-2 products while a microwave activation at the same temperature gave a 49/51 mixture. Epoxidation and indole alkylation from **9g** and **9h** under the same conditions as in route 2 gave the final products **11l** and **11m** (Scheme 3).

### 2.2. X-ray Structural Studies

**Figure 2:**
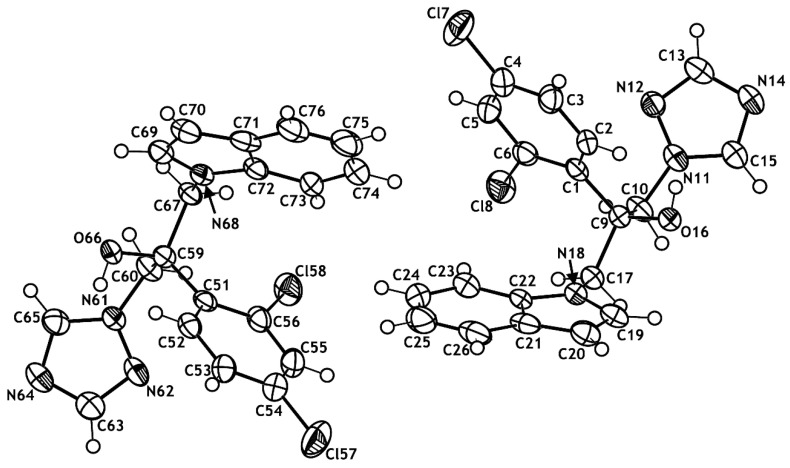
View of the crystal structure of (*R*)-**8g** with our numbering scheme, displacement ellipsoids are drawn at the 30% probability level.

**Figure 3:**
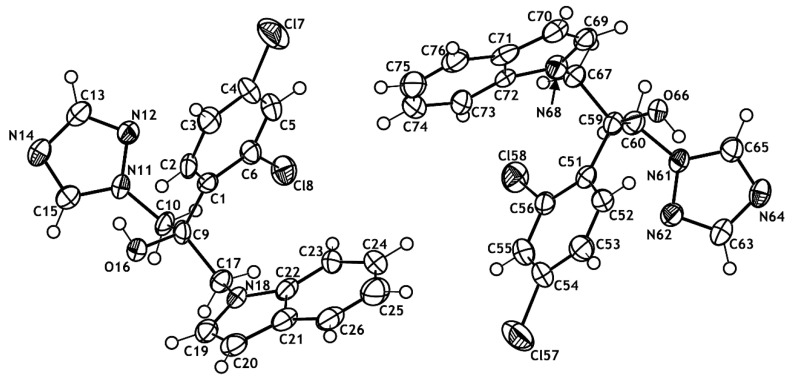
View of the crystal structure of (*S*)-**8g** with our numbering scheme, displacement ellipsoids are drawn at the 30% probability level.

In order to confirm the absolute configuration of the synthesized compounds **8g**, X-ray structure analyses for the two derivatives were performed. The atom labeling and thermal ellipsoids of (*+*)-**8g** and (−)-**8g** are shown in Figure 2 and Figure 3. Two independent molecules, designated as A and B, were found in the asymmetric crystallographic unit of (*+*)-**8g** and (−)-**8g**. The configuration of (*+*)-**8g** and (−)-**8g** was determined by observing and calculating the *F*(+)/*F*(−) ratios of Bijvoet pairs with the mean *F* value of each independent reflection. Based on the results, the absolute configuration at C-9 and C-59 in (*+*)-**8g** was determined to be *R*. The (−)-**8g** enantiomer was found with the *S*-absolute configuration.

### 2.3. Biological Results

#### 2.3.1. In Vitro Anti-*Candida* Activity

All anti-*Candida* activities of propanol derivatives **8a**–**g** and **11a**–**m** are presented in Table 1. Concerning the expression of the results during the evaluation of the compounds, we deliberately chose to calculate a more selective minimal inhibitory concentration (MIC = IC_80_, µg/mL) than an IC_50_ in order to highlight some very active compounds. Concerning the 1*H*-1,2,4-triazole sub-series, substitutions of the phenyl ring by halogens greatly impacted anti-*C. albicans* activities. The 4-trifluoromethylated **8f**, 4-chlorinated **11b**, 4-brominated **11c**, and 2,4-difluorinated **11d** compounds had activities comparable with FLC (MIC = 0.020 µg/mL on *C. albicans* CA98001), while the 4-fluorinated derivative **11a** was less active (MIC = 0.210 µg/mL). The most favorable substitution corresponded to the 2,4-dichlorinated phenyl ring for compound **8g**. The asymmetric carbon of the propanol chain also played a very important role in the activity against *C. albicans*. Indeed, activity of the (+)-(*R*)-enantiomer was similar to FLC (MIC = 0.023 µg/mL), whereas the (−)-(*S*)-enantiomer had an action comparable with the racemic mixture (MIC values of 0.000256 and 0.000259 µg/mL, respectively).

In the imidazole sub-series, there was little difference in activity depending on the substitution of the phenyl ring because all compounds had activities similar to that of FLC except for the 2,4-difluorinated derivative **8d**, which was 57 times more active (MIC = 0.00035 µg/mL). There was no correlation between the imidazole and 1*H*-1,2,4-triazole series according to the substitution of the phenyl ring. Compounds of 1*H*-1,2,3-triazole and 2*H*-1,2,3-triazole series with 2,4-dichlophenyl substitution **11l**–**m** were totally inactive. This pharmacomodulation of the azolyl unit highlighted the necessity of the 1*H*-1,2,4-triazole or imidazole moiety to promote activity against *C. albicans*. The inactivity of 1*H*-1,2,3-triazole and 2*H*-1,2,3-triazole derivatives could be explained by the impossibility of these rings to coordinate with the iron atom of CYP51.

The last work of pharmacomodulation focused on the presence of the methyl group in position 2 and/or 3 of the indole ring. With a 2,4-difluorinated phenyl group, the presence of a methyl at position 2 on the indole ring did not change the activity (**11f**, MIC = 0.022 µg/mL); on the other hand, the introduction of a methyl at position 3 enhanced the activity level (**11h**, MIC = 0.011 µg/mL). In the case of two methyl groups at positions 2 and 3, the activity was reduced (**11j**, MIC = 0.157 µg/mL). The activities of 2,4-dichloro derivatives were generally better than those encountered with 2,4-difluorinated products. However, none of the proposed pharmacomodulations retained the activity obtained with the unsubstituted indole. In this case, the inactivity of the 2,3-dimethylindole unit was even more pronounced. The conjugated steric effect of the methyl groups on positions 2 and 3 on the indole ring may have been the cause for this decrease in activity, by imposing an unfavorable position of the indole in the active site of the enzyme.

Overall, it emerged that the synthesized compounds showed activity against *C. albicans* comparable with or greater than fluconazole. For example, difluoro derivatives (**8d** and **11h**) and dichloro derivatives (**8g** and **11i**) were the most active derivatives. In the particular case of compound **8g**, (±)-**8g** and its (−)-(*S*) enantiomer were ≈80 times more active than FLC against *C. albicans* CA98001.

The activity spectrum on *Candida* species (Table 2) shows that compound **8g** and more particularly its (*S*)-enantiomer maintained a better activity than FLC against the various strains tested, especially on those naturally resistant to FLC. For example, on *C. krusei*, the MIC values of (*S*)-**8g** were between 0.167 and 0.039 µg/mL.

#### 2.3.2. Toxicity of Selected Compounds on Human Fetal Lung Fibroblast (MRC-5) Cells

The in vitro toxicity of compounds **8a**–**g** and **11a**–**k** was evaluated on MRC-5 cells. The results are shown in Table 3. In the imidazole sub-series (compounds **8a**–**e** and **11e**), the cytotoxicity (IC_50_ = 31–50 µM) was approximatively 1.4 to 2.2 times higher than that of ketoconazole (KTC) (IC_50_ = 69.1 µM). Comparison of this sub-series with the 1,2,4-triazole sub-series (unsubstituted indole derivatives **11a**, **11b**, and **11d**) has shown that the triazole moiety decreased significantly the cytotoxicity (IC_50_ > 100 µM), except for the 4-bromo derivative **11c** (IC_50_ = 38 µM), **8g**, and its enantiomers. Indeed, the cytotoxicity values of **8g**, (+)-(*R*)-**8g**, and (−)-(*S*)-**8g** (IC_50_ = 35 µM [28], 32, and 30 µM, respectively) were two times higher than that of KTC and at least three times higher than those of FLC (IC_50_ > 100 µM).

The presence of a methyl group in position 2 and/or 3 on the indole ring (derivatives **11f**–**k**) was compared with the unsubstituted indole derivatives. With a 2,4-difluorinated **11f** phenyl ring (≈ 190 µM), the presence of a methyl at position 2 did not change the cytotoxicity on MRC-5 cells when compared to compound **11d** (≈ 197 µM). On the contrary, with a 2,4-dicholorinated **11g** phenyl ring (= 157 µM), a methyl group at position 2 decreased the cytotoxicity on MRC-5 cells when compared to compound **8g** (= 35 µM). On the other hand, the presence of a methyl group at position 3 led to a two-fold increase in cytotoxicity for **11h** (2,4-difluoro derivative, IC_50_ = 105.4 µM) and for **11i** as well (2,4-dichloro derivative, IC_50_ = 19 µM). In the sub-series of 2,3-dimethyl derivatives, the cytotoxicity was increased with **11j** (2,4-difluoro derivative, IC_50_ = 25 µM) compared with compound **11d** (≈ 197 µM). In the case of **11k** (2,4-dichloro derivative, IC_50_ ≈ 97 µM), the cytotoxicity on MRC-5 cells was decreased compared with compound **8g** (= 35 µM). While the presence of a methyl group on the indole ring globally increased the cytotoxicity of the 2,4-difluoro derivatives **11h** and **11j**, the introduction of a methyl group in position 2 into 2,4-dichlorinated products seemed to give less cytotoxic compounds (e.g. **11g**, **11k**).

#### 2.3.3. Inhibitory Activity on Cytochrome P450 Enzymes

Triazole derivatives **11d** (2,4-difluoro) and **8g** (2,4-dichloro) were further investigated for their potential to inhibit diverse cytochrome P450 enzymes. Among our panel, four steroidogenic enzymes involved in the biosynthesis of active steroid hormones were selected. This includes 11β-hydroxylase (CYP11B1), aldosterone synthase (CYP11B2), 17α-hydroxylase/17,20-lyase (CYP17), and aromatase (CYP19). Another enzyme, retinoic acid 4-hydroxylase (CYP26A1), was also used to check the selectivity of compounds **11d** and **8g**. Due to the presence of 1*H*-1,2,4-triazole, both compounds could coordinate their *N*-4 atom with the heme iron of these enzymes. In addition, it is the reason that the majority of reference compounds used in our assays (Table 4) were azole agents such as anastrozole, letrozole, liarozole, BW19 (4-(6-methoxy-1-methyl-3,4-dihydro-naphthalen-2-ylmethyl)-1*H*-imidazole) [29], and KTC. Aminoglutethimide (AG) and fadrozole possess other nitrogen moieties such as amino and nitrile groups, respectively.

First, compounds **11d**, **8g**, and its enantiomers were tested against CYP19 at 36 µM. Only **11d** (IC_50_ 3.58 µM), **8g**, and its enantiomers (+)-(*R*) and (−)-(*S*) (% inhibition of 27, 51, and 72, respectively) were weak inhibitors of CYP19. Some studies described inhibitory activities of FLC against CYP19 (IC_50_ = 26.8, >140 or = 300 µM, depending on the method used [30,31]). Comparing (−)-(*S*)-**8g** with letrozole, the most potent non-steroidal inhibitor (IC_50_ = 0.025 nM), there was no significant activity for our antifungal agent against CYP19. In the opposite way, all works undertaken around the indole and the design of non-steroidal aromatase inhibitors [18,19,32,33,34,35,36] helped us to design our first antifungal azolyl-substituted indoles [22,23].

For CYP17, all compounds tested (**11d**, **8g**, and its enantiomers) were either inactive or very weak inhibitors. For example, **8g** gave an inhibitory effect below 10% at 2.5 µM. The range was very significant with BW19 and KTC, with IC_50_ values of 0.15 and 4.5 µM, respectively.

The enantiomers of **8g** were evaluated for inhibitory activity against retinoic acid 4-hydroxylase (CYP26A1) using a cellular assay. The standards for comparison in the CYP26A1 assay were the broad spectrum CYP inhibitor KTC and liarozole. The (+)-(*R*)-**8g** was a very weak inhibitor of CYP26A1 (IC_50_ = 34 µM), while the (−)-(*S*)-**8g** enantiomer also displayed weak inhibitory activity against CYP26A1 (IC_50_ = 18 µM) compared with KTC (IC_50_ = 10 µM) and liarozole (IC_50_ = 7 µM).

Racemic compound **8g** and its enantiomers were also tested for their potential inhibitory effect against CYP11B1 and CYP11B2. **8g** showed no inhibitory activity against CYP11B1 and only a slight inhibitory effect against CYP11B2 (at high concentrations). Both enantiomers showed no inhibitory activity against CYP11B1. As mentioned before [37], KTC was a strong inhibitor of CYP11B1; on the other hand, FLC was a minor inhibitor. Only (+)-(*R*)-**8g** showed a slight inhibitory effect against CYP11B2 (at high concentrations) while KTC exerted 100% inhibition in another similar study [38].

### 2.4. Molecular Modeling Studies

#### 2.4.1. Molecular Docking

**Figure 4:**
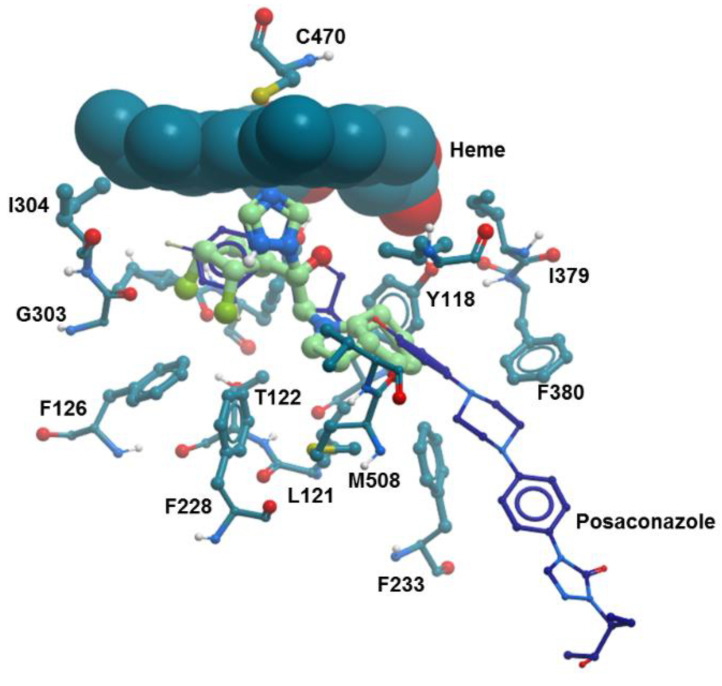
Predicted binding pose of (*S*)-**8g** (light green) using CaCYP51.

**Figure 5:**
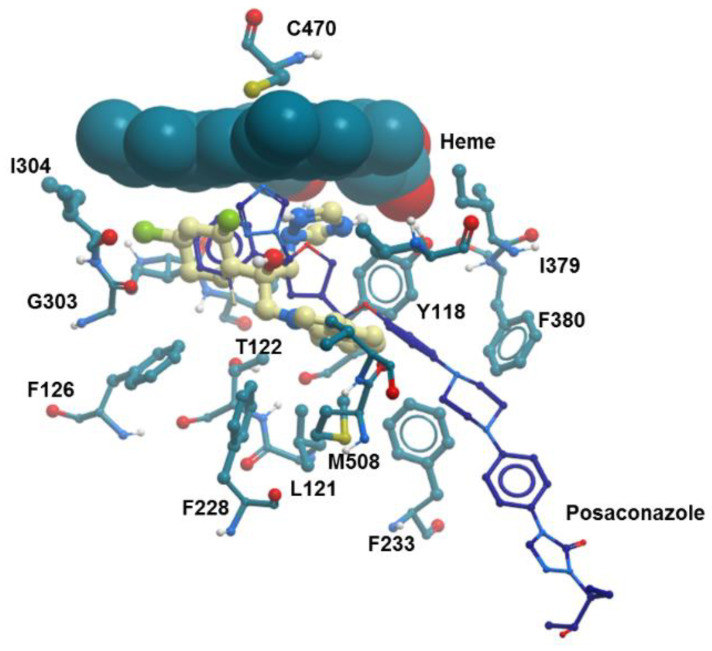
Predicted binding pose of (*R*)-**8g** (cream) using CaCYP51.

The predicted binding pose of (*S*)-**8g** (Figure 4) was to some degree consistent with the binding conformation of co-crystallized posaconazole in the Protein Data Bank (PDB) structure (PDB ID: 5FSA [39]) with the nitrogen atom in the triazole ring interacting with the Fe atom in the heme group. However, the predicted binding pose of (*R*)-**8g** (Figure 5) was not consistent with the posaconazole pose in its co-crystal structure where the triazole ring was more parallel with the heme group, rather than perpendicular to the heme, that is, a less favorable angle and subsequently a weaker binding interaction would be expected. Therefore, the (*S*)-**8g** generated a better docking score and more credible binding pose compared with (*R*)-**8g**, which is consistent with the biological experiments described in 2.3.1.

#### 2.4.2. Molecular Dynamics Simulation

In order to confirm the first results obtained by molecular docking, a molecular dynamics (MD) approach was also applied to CaCYP51-azole complexes.

The (*R*)- and (*S*)-enantiomers of compound **8g** were docked with the CaCYP51 crystal structure (PDB 5FSA [39]) using molecular operating environment (MOE) software [40], and the resulting CaCYP51 (*R*)-**8g** and (*S*)-**8g** ligand-protein complexes subjected to 100 ns MD simulations using the Desmond programme of Maestro [41].

**Figure 6:**
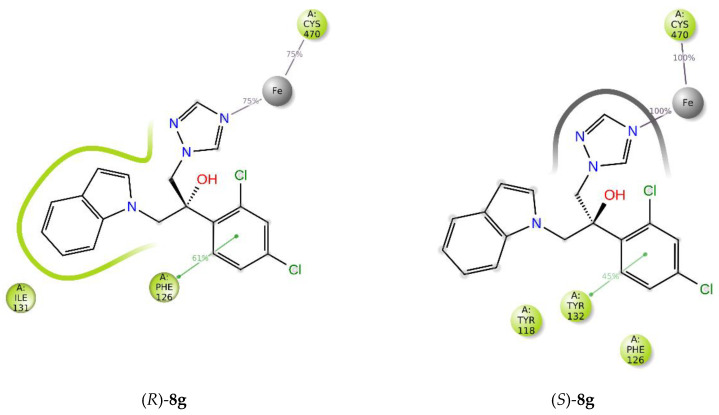
A schematic of detailed ligand atom interactions with the protein residues. Interactions that occur more than 30.0% of the simulation time in the selected trajectory (0.00 through 100.00 ns) are shown.

**Figure 7:**
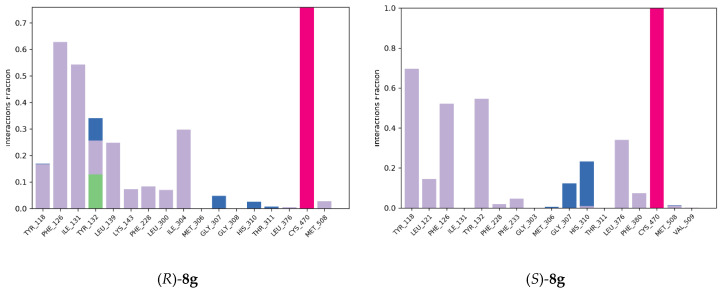
A schematic of detailed ligand atom interactions of (*R*)-**8g** and (*S*)-**8g** with the protein residues of wild-type CaCYP51 protein. Interactions that occur more than 30.0% of the simulation time in the selected trajectory (0 through 100 ns) are shown. (Hydrophobic (purple), water bridges (blue), H-bonds (green), ionic (pink)).

Both enantiomers formed primarily hydrophobic interactions including Tyr118, Phe126, and Tyr132 for both enantiomers, Ile304 for (*R*)-**8g**, and Leu376 for (*S*)-**8g** (Figure 6 and Figure 7). In both enantiomers the dichlorophenyl ring forms a π–π stacking interaction, with Phe126 and Tyr132 for the (*R*)-**8g** and (*S*)-**8g** enantiomer, respectively. Of note is the interaction of the triazole N with the heme iron, which occurred for 75% of the simulation time for (*R*)-**8g** and 100% of the simulation time for (*S*)-**8g**, suggesting improved binding of the (*S*)-enantiomer with the heme active site.

**Figure 8:**
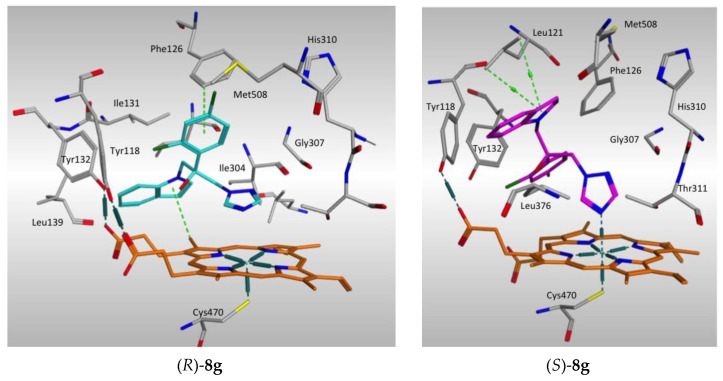
3D images of (*R*)-**8g** (cyan) and (*S*)-**8g** (magenta) ligand-CaCYP51 protein complexes after MD simulation, showing key binding amino acids and positioning of the triazole ring above the heme (orange).

The reason for the reduced binding interaction of the (*R*)-enantiomer of **8g** can be seen from the 3D image after MD simulation (Figure 8). For optimal binding of the triazole nitrogen with the heme iron, the triazole should have been positioned perpendicular to the heme as observed for the (*S*)-**8g** enantiomer; however, the triazole of the (*R*)-**8g** enantiomer was positioned parallel to the heme, reducing optimal binding. The unfavorable position of the (*R*)-enantiomer triazole also increased the distance between the triazole N and the heme iron to 3.11 Å compared with 2.43 Å for the optimally positioned (*S*)-enantiomer.

**Figure 9:**
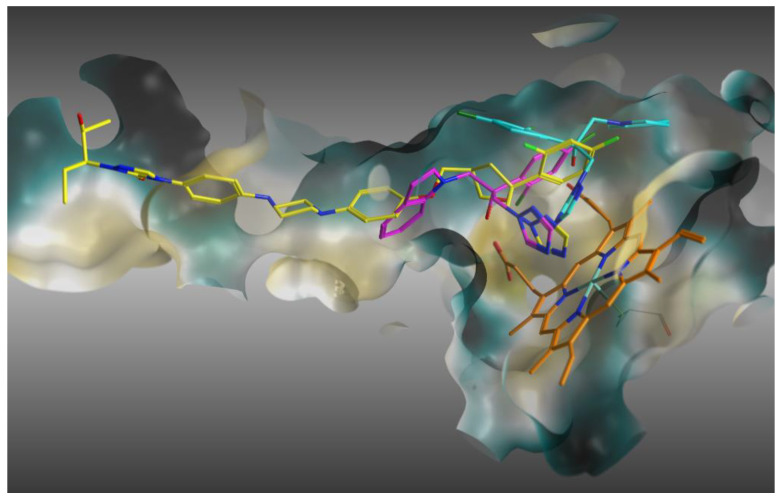
CaCYP51-posaconazole complex (PDB 5FSA) with posaconazole (yellow) positioned along the access channel and above the heme (orange). (*R*)-**8g** (cyan) and (*S*)-**8g** (magenta) are aligned after MD simulations.

The overlap of both enantiomers of **8g** and posaconazole shows that the (*S*)-enantiomer aligned with the positioning of posaconazole within both the heme and access channel binding sites, whereas for the (*R*)-enantiomer the dichlorophenyl was outside the small aryl binding pocket and the indole was directed away from the access channel (Figure 9).

## 3. Materials and Methods

### 3.1. Chemistry

The microwave reactor was a monomode system (Discover, CEM) with focused waves operating at 2.45 GHz. Melting points were determined on an Electrothermal IA9300 melting point digital apparatus and were reported uncorrected. Infrared (IR) spectra were obtained in KBr pellets or neat liquid films with a Perkin–Elmer Paragon FTIR 1000 PC spectrometer. ^1^H and ^13^C-NMR spectra were recorded on a Bruker AC 250 or AVANCE 400 spectrometer in DMSO-*d_6_* as solvent. Chemical shifts (δ) were reported in part per million (ppm) relative to tetramethylsilane as internal standard. The splitting pattern abbreviations are as follows: s, singlet; d, doublet; t, triplet; q, quartet; m, multiplet; dd, doublet of doublet; td, triplet of doublet. Coupling constants J were given in Hz. Mass spectral (MS) analysis was performed on a quadrupole H.P. 5889A instrument using EI mode at 70 eV. Microanalyses were performed on a Perkin–Elmer CHN 240 apparatus. All reactions were monitored by TLC, using 0.25 mm-thick precoated silica gel plates (E. Merck, Darmstadt, Germany). Compounds were purified by column chromatography using silica gel 60 as a stationary phase. All common chemicals and solvents utilized were reagent grade and purchased from Sigma–Aldrich (Saint Quentin, France).

The synthesis of key intermediates **9a**–**e** and **10a**–**e** was previously described by IICiMed (Nantes) [27]. 1-Hydroxymethyl-1*H*-benzotriazole (**2**), 1-chloromethyl-1*H*-benzotriazole (**3**), and 1-*(*1*H-*benzotriazol-1-yl-methyl*)-*1*H-*indole (**4**) were previously published [42,43] and some spectral data were added to be as precise as possible.

### 3.2. Synthesis of Compounds ***8a***–***g*** (Route 1)

#### 3.2.1. Procedure for the Synthesis of 1-Hydroxymethyl-1*H*-benzotriazole (**2**)

A solution of 1*H*-benzotriazole (**1**) (10 g, 83.94 mmol) in formaldehyde (37% in aqueous solution, 6.81 mL, 83.94 mmol) was stirred at rt for 5 min. The precipitate was filtered and recrystallized from THF. 1-Hydroxymethyl-1*H*-benzotriazole (**2**) was obtained (94% yield): White powder; Rf = 0.45 (dichloromethane /EtOH: 19/1); mp: 148–150 °C (148–151 °C, lit. [42]); IR (KBr): ν 3192 (s, OH), 1622, 1510 cm^−1^; ^1^H NMR (DMSO-*d_6_*): δ 8.11 (d, 1H, *J* = 8.3 Hz, H_4_), 7.95 (d, 1H, *J* = 8.3 Hz, H_7_), 7.46–7.62 (m, 2H, H_5_, H_6_), 7.27 (t, 1H, *J* = 7.7 Hz, OH), 6.07 (d, 2H, *J* = 7.7 Hz, CH_2_).

#### 3.2.2. Procedure for the Synthesis of 1-Chloromethyl-1*H*-benzotriazole (**3**)

1-Hydroxymethyl-1*H*-benzotriazole (**2**) (8.91 g, 59.7 mmol) was cooled at 0 °C and thionyl chloride (26 mL, 360 mmol) was added dropwise. Then the mixture was stirred and refluxed for 1 h and the volatile fraction was evaporated under reduced pressure. The residue was dissolved in MeOH and filtered at rt. 1-Chloromethyl-1*H*-benzotriazole (**3**) was obtained (93% yield): White powder; Rf = 0.74 (dichloromethane /EtOH: 19/1); mp: 136–138 °C (136–138 °C, lit. [42]); IR (KBr): ν 1612, 1506, 1095 cm^−1^; ^1^H NMR (DMSO-*d_6_*): δ 8.19 (d, 1H, *J* = 8.3 Hz, H_4_), 8.06 (d, 1H, *J* = 8.4 Hz, H_7_), 7.52–7.76 (m, 2H, H_5_, H_6_), 6.93 (s, 2H, CH_2_).

#### 3.2.3. Procedure for the Synthesis of 1-(1*H*-Benzotriazol-1-yl-methyl)-1*H*-indole (**4**)

Sodium hydride (0.73 g, 30.3 mmol) was dissolved in DMSO (30 mL) and 1*H*-indole (3.23 g, 27.6 mmol) was added portionwise and the mixture was stirred at rt for 1 h. Then 1-chloromethyl-1*H*-benzotriazole (**3**) (4.62 g, 27.6 mmol) was added and the reaction mixture stirred for 2 h. At the end of this period, the mixture was diluted with H_2_O (30 mL) and extracted with EtOAc (3 × 30 mL). The organic layer was washed with brine (30 mL), dried over anhydrous sodium sulfate, and the volatile fraction was evaporated under reduced pressure. The residue was crystallized from diisopropyl ether. 1-(1*H*-Benzotriazol-1-yl-methyl)-1*H*-indole (**4**) was obtained (80% yield): White powder; Rf = 0.35 (dichloromethane); mp: 176–178 °C (176–178 °C, lit. [43]); IR (KBr): ν 1616, 1511, 1349 cm^−1^; ^1^H NMR (DMSO-*d_6_*): δ 8.15 (d, 1H, *J* = 8.4 Hz, H_4′_), 8.07 (d, 1H, *J* = 8.4 Hz, H_7′_), 7.86 (d, 1H, *J* = 7.2 Hz, H_4_), 7.85 (d, 1H, *J* = 3.3 Hz, H_2_), 7.56 (d, 1H, *J* = 7.9 Hz, H_7_), 7.41–7.70 (m, 2H, H_5′_, H_6′_), 7.28 (s, 2H, CH_2_), 7.24 (dd, 1H, *J* = 7.9 Hz, *J* = 7.2 Hz, H_6_), 7.09 (dd, 1H, *J* = *J* = 7.2 Hz, H_5_), 6.56 (d, 1H, *J* = 3.3 Hz, H_3_).

#### 3.2.4. General Procedure for the Synthesis of Benzotriazole Derivatives **5a**–**f**

1-(1*H*-Benzotriazol-1-yl-methyl)-1*H*-indole (**4**) (1.88 g, 7.57 mmol) in THF (50 mL) was cooled to –78 °C under argon, and n-BuLi (1.6 M in THF, 5.67 mL, 9.1 mmol) was added dropwise, then the mixture was stirred at –78 °C for 1 h. At the end of this period, the corresponding ethyl benzoate (9.46 mmol) was added dropwise and the mixture was slowly warmed to rt over a period of 12 h. Then saturated aqueous ammonium chloride solution (35 mL) was added, the mixture was diluted with H_2_O (30 mL), and extracted with diethyl ether (3 × 40 mL). The organic layer was washed with brine (40 mL), dried over anhydrous sodium sulfate, and the volatile fraction was evaporated under reduced pressure. The residue was purified by silica gel column chromatography (dichloromethane).

1-[(1*H*-Benzotriazol-1-yl)(4-fluorobenzoyl)methyl]-1*H*-indole (**5a**). White powder (61% yield); Rf = 0.29 (dichloromethane); mp: 121–123 °C; IR (KBr): ν 1701 (s, C=O), 1594, 1304 cm^−1^; ^1^H NMR (DMSO-*d_6_*): δ 9.45 (s, 1H, CH), 8.09–8.12 (m, 1H, H_4′_), 8.03–8.07 (m, 2H, H_2″_, H_6″_), 8.01 (d, 1H, *J* = 8.2 Hz, H_7′_), 7.90 (d, 1H, *J* = 8.2 Hz, H_4_), 7.60–7.67 (m, 1H, H_6′_), 7.56–7.59 (m, 1H, H_7_), 7.56 (d, 1H, *J* = 3.4 Hz, H_2_), 7.44–7.50 (m, 1H, H_5′_), 7.35–7.42 (m, 2H, H_3″_, H_5″_), 7.22 (dd, 1H, *J* = 8.2 Hz, *J* = 7.1 Hz, H_6_), 7.12 (dd, 1H, *J* = 8.2 Hz, *J* = 7.1 Hz, H_5_), 6.60 (d, 1H, *J* = 3.4 Hz, H_3_).

1-[(1*H*-Benzotriazol-1-yl)(4-chlorobenzoyl)methyl]-1*H*-indole (**5b**). White powder (61% yield); Rf = 0.30 (dichloromethane); mp: 184–185 °C; IR (KBr): ν 1712 (s, C=O), 1587, 1323 cm^−1^; ^1^H NMR (DMSO-*d_6_*): δ 9.46 (s, 1H, CH), 8.11 (d, 1H, *J* = 8.1 Hz, H_4′_), 8.00 (d, 1H, *J* = 8.2 Hz, H_7′_), 8.00 (d, 2H, *J* = 8.5 Hz, H_2″_, H_6″_), 7.90 (d, 1H, *J* = 8.3 Hz, H_4_), 7.64 (d, 2H, *J* = 8.5 Hz, H_3″_, H_5″_), 7.60–7.64 (m, 1H, H_5′_), 7.56–7.62 (m, 1H, H_7_), 7.57 (d, 1H, *J* = 3.1 Hz, H_2_), 7.47 (dd, 1H, *J* = 8.2 Hz, *J* = 7.9 Hz, H_6′_), 7.26 (dd, 1H, *J* = 7.6 Hz, *J* = 7.3 Hz, H_6_), 7.13 (dd, 1H, *J* = 8.3 Hz, *J* = 7.3 Hz, H_5_), 6.61 (d, 1H, *J* = 3.1 Hz, H_3_).

1-[(1*H*-Benzotriazol-1-yl)(4-bromobenzoyl)methyl]-1*H*-indole (**5c**). White powder (50% yield); Rf = 0.40 (dichloromethane); mp: 156–157 °C; IR (KBr): ν 1710 (s, C=O), 1583, 1323 cm^−1^; ^1^H NMR (DMSO-*d_6_*): δ 9.46 (s, 1H, CH), 8.12 (d, 1H, *J* = 8.3 Hz, H_4′_), 8.03 (d, 1H, *J* = 8.4 Hz, H_7′_), 7.92 (d, 2H, *J* = 8.6 Hz, H_2″_, H_6″_), 7.89 (d, 1H, *J* = 7.2 Hz, H_4_), 7.78 (d, 2H, *J* = 8.6 Hz, H_3″_, H_5″_), 7.65 (dd, 1H, *J* = 8.4 Hz, *J* = 8.0 Hz, H_6′_), 7.57 (d, 1H, *J* = 3.3 Hz, H_2_), 7.56–7.60 (m, 1H, H_7_), 7.47 (dd, 1H, *J* = 8.3 Hz, *J* = 8.0 Hz, H_5′_), 7.26–7.30 (m, 1H, H_6_), 7.16 (dd, 1H, *J* = *J* = 7.2 Hz, H_5_), 6.61 (d, 1H, *J* = 3.3 Hz, H_3_).

1-[(1*H*-Benzotriazol-1-yl)(4-trifluoromethylbenzoyl)-methyl]-1*H*-indole (**5d**). Yellow powder (75% yield); mp: 175–177 °C; IR (KBr): ν 1711 (s, C=O), 1598, 1330 cm^−1^; ^1^H NMR (DMSO-*d_6_*): δ 9.55 (s, 1H, CH), 8.21 (d, 2H, *J* = 7.9 Hz, H_2″_, H_6″_), 8.12 (d, 1H, *J* = 8.6 Hz, H_4′_), 8.08 (d, 1H, *J* = 8.6 Hz, H_7′_), 7.96 (d, 2H, *J* = 7.9 Hz, H_3″_, H_5″_), 7.93–7.96 (m, 1H, H_4_), 7.67 (dd, 1H, *J* = *J* = 8.6 Hz, H_6′_), 7.58 (d, 1H, *J* = 3.1 Hz, H_2_), 7.58–7.61 (m, 1H, H_7_), 7.48 (dd, 1H, *J* = *J* = 8.6 Hz, H_5′_), 7.28 (dd, 1H, *J* = *J* = 7.0 Hz, H_6_), 7.14 (dd, 1H, *J* = *J* = 7.0 Hz, H_5_), 6.62 (d, 1H, *J* = 3.1 Hz, H_3_).

1-[(1*H*-Benzotriazol-1-yl)(2,4-difluorobenzoyl)methyl]-1*H*-indole (**5e**). White powder (41% yield); Rf = 0.26 (dichloromethane); mp: 111–113 °C; IR (KBr): ν 1705 (s, C=O), 1580, 1334 cm^−1^; ^1^H NMR (DMSO-*d_6_*): δ 9.02 (s, 1H, CH), 8.26–8.77 (m, 1H, H_6″_), 8.10 (d, 1H, *J* = 8.5 Hz, H_4′_), 8.02 (d, 1H, *J* = 8.5 Hz, H_7′_), 7.78 (d, 1H, *J* = 8.6 Hz, H_4_), 7.62–7.68 (m, 1H, H_6′_), 7.61 (d, 1H, *J* = 3.1 Hz, H_2_), 7.57 (d, 1H, *J* = 7.9 Hz, H_7_), 7.44–7.53 (m, 1H, H_5′_), 7.44–7.47 (m, 1H, H_3″_), 7.41–7.44 (m, 1H, H_5″_), 7.22 (dd, 1H, *J* = 7.9 Hz, *J* = 7.6 Hz, H_6_), 7.11 (dd, 1H, *J* = 8.6 Hz, *J* = 7.6 Hz, H_5_), 6.59 (d, 1H, *J* = 3.1 Hz, H_3_).

1-[(1*H*-Benzotriazol-1-yl)(2,4-dichlorobenzoyl)methyl]-1*H*-indole (**5f**). White powder (51% yield); Rf = 0.34 (dichloromethane); mp: 141–142 °C; IR (KBr): ν 1714 (s, C=O), 1582, 1375 cm^−1^; ^1^H NMR (DMSO-*d_6_*): δ 9.34 (s, 1H, CH), 8.14 (d, 1H, *J* = 8.3 Hz, H_4′_), 8.09 (d, 1H, *J* = 8.3 Hz, H_7′_), 7.85 (d, 1H, *J* = 8.2 Hz, H_4_), 7.71–7.76 (m, 1H, H_6″_), 7.68–7.71 (m, 1H, H_6′_), 7.59 (d, 1H, *J* = 3.1 Hz, H_2_), 7.41–7.50 (m, 2H, H_7,_ H_5′_), 7.31–7.39 (m, 1H, H_3″_), 7.26 (dd, 1H, *J* = 7.6 Hz, *J* = 7.3 Hz, H_6_), 7.05–7.15 (m, 1H, H_5″_), 6.99 (dd, 1H, *J* = 8.2 Hz, *J* = 7.3 Hz, H_5_), 6.44 (d, 1H, *J* = 3.1 Hz, H_3_).

#### 3.2.5. General Procedure for the Synthesis of *N*-Substituted-1*H*-indoles **6a**–**f**

The corresponding 1-[(1*H*-benzotriazol-1-yl)(halogenobenzoyl)methyl]-1*H*-indole **5a**–**f** (3.00 mmol) in ethanol (15 mL) and THF (15 mL) was stirred at rt. Then acetic acid (3 mL) and zinc (0.98 g, 15 mmol) were added, and the reaction mixture was stirred at 35 °C for 5 h in an ultrasonic bath. The mixture was filtered on Celite 545 and the filtrate was evaporated under reduced pressure. The residue was diluted with H_2_O (40 mL) and extracted with EtOAc (3 × 40 mL), then the organic layer was washed with H_2_O (40 mL), dried over anhydrous sodium sulfate, and the volatile fraction was evaporated under reduced pressure. The residue was crystallized from diisopropyl ether.

*N*-(4-Fluorobenzoylmethyl)-1*H*-indole (**6a**). White powder (57% yield); Rf = 0.61 (dichloromethane); mp: 153–154 °C; IR (KBr): ν 1691 (s, C=O), 1597, 1325 cm^−1^; ^1^H NMR (DMSO-*d_6_*): δ 8.19–8.24 (m, 2H, H_2′_, H_6′_), 7.60 (d, 1H, *J* = 7.4 Hz, H_4_), 7.48–7.51 (m, 2H, H_3′_, H_5′_), 7.37–7.44 (m, 2H, H_2,_ H_7_), 7.02–7.13 (m, 2H, H_5,_ H_6_), 6.52 (d, 1H, *J* = 3.1 Hz, H_3_), 5.93 (s, 2H, CH_2_).

*N*-(4-Chlorobenzoylmethyl)-1*H*-indole (**6b**). White powder (66% yield); Rf = 0.62 (dichloromethane); mp: 156–157 °C; IR (KBr): ν 1692 (s, C=O), 1586, 1399 cm^−1^; ^1^H NMR (DMSO-*d_6_*): δ 8.12–8.15 (m, 1H, H_4_), 7.72 (d, 2H, *J* = 8.6 Hz, H_2′_, H_6′_), 7.58–7.60 (m, 1H, H_7_), 7.37 (d, 2H, *J* = 8.6 Hz, H_3′_, H_5′_), 7.35–7.40 (m, 1H, H_2_), 7.04–7.11 (m, 2H, H_5,_ H_6_), 6.50 (d, 1H, *J* = 3.1 Hz, H_3_), 5.93 (s, 2H, CH_2_).

*N*-(4-Bromobenzoylmethyl)-1*H*-indole (**6c**). White powder (66% yield); Rf = 0.60 (dichloromethane); mp: 185–186 °C; IR (KBr): ν 1689 (s, C=O), 1582, 1384 cm^−1^; ^1^H NMR (DMSO-*d_6_*): δ 8.07 (d, 2H, *J* = 8.5 Hz, H_2′_, H_6′_), 7.87 (d, 2H, *J* = 8.5 Hz, H_3′_, H_5′_), 7.60 (d, 1H, *J* = 7.3 Hz, H_4_), 7.40 (d, 1H, *J* = 8.2 Hz, H_7_), 7.35 (d, 1H, *J* = 3.1 Hz, H_2_), 7.12 (dd, 1H, *J* = 8.2 Hz, *J* = 7.3 Hz, H_6_), 7.05 (dd, 1H, *J = J =* 7.3 Hz, H_5_), 6.52 (d, 1H, *J* = 3.1 Hz, H_3_), 5.94 (s, 2H, CH_2_).

*N*-(4-Trifluoromethylbenzoylmethyl)-1*H*-indole (**6d**). White powder (45% yield); mp: 182–185 °C; IR (KBr): ν 1690 (s, C=O), 1580, 1382 cm^−1^; ^1^H NMR (DMSO-*d_6_*): δ 8.33 (d, 2H, *J* = 7.6 Hz, H_2′_, H_6′_), 8.03 (d, 2H, *J* = 7.6 Hz, H_3′_, H_5′_), 7.61 (d, 1H, *J* = 8.0 Hz, H_4_), 7.43 (d, 1H, *J* = 8.0 Hz, H_7_), 7.38 (d, 1H, *J* = 3.1 Hz, H_2_), 7.13 (dd, 1H, *J = J =* 8.0 Hz, H_5_), 6.93 (dd, 1H, *J = J =* 8.0 Hz, H_6_), 6.54 (d, 1H, *J* = 3.1 Hz, H_3_), 6.02 (s, 2H, CH_2_).

*N*-(2,4-Difluorobenzoylmethyl)-1*H*-indole (**6e**). White powder (60% yield); Rf = 0.39 (dichloromethane); mp: 101–103 °C; IR (KBr): ν 1698 (s, C=O), 1587, 1379 cm^−1^; ^1^H NMR (DMSO-*d_6_*): δ 8.03–8.12 (m, 1H, H_6′_), 7.58–7.60 (m, 1H, H_4_), 7.43 (d, 1H, *J* = 7.3 Hz, H_7_), 7.35–7.41 (m, 2H, H_2,_ H_5′_), 7.31–7.35 (m, 1H, H_3′_), 7.02–7.11 (m, 2H, H_5,_ H_6_), 6.51–6.52 (m, 1H, H_3_), 5.79 (s, 2H, CH_2_).

*N*-(4-Dichlorobenzoylmethyl)-1*H*-indole (**6f**). White powder (46% yield); Rf = 0.64 (dichloromethane); mp: 98–99 °C; IR (KBr): ν 1704 (s, C=O), 1580, 1367 cm^−1^; ^1^H NMR (DMSO-*d_6_*): δ 8.04 (d, 1H, *J* = 8.5 Hz, H_6′_), 7.83 (d, 1H, *J* = 1.4 Hz, H_3′_), 7.66–7.70 (m, 1H, H_5′_), 7.60 (d, 1H, *J* = 7.3 Hz, H_4_), 7.42 (d, 1H, *J* = 7.9 Hz, H_7_), 7.38 (d, 1H, *J* = 3.1 Hz, H_2_), 7.16 (dd, 1H, *J* = 7.9 Hz, *J* = 7.0 Hz, H_5_), 7.07 (dd, 1H, *J* = 8.5, *J* = 7.0 Hz, H_6_), 6.52 (d, 1H, *J* = 3.1 Hz, H_3_), 5.77 (s, 2H, CH_2_).

#### 3.2.6. General Procedure for the Synthesis of Oxiranes **7a**–**f**

Trimethylsulfoxonium iodide (0.44 g, 2.02 mmol) and sodium hydroxide (6 g, 150 mmol, 48% in aqueous solution) were added to a solution of the corresponding *N*-substituted indole **6a**–**f** (1.41 mmol) in dichloromethane (5 mL). Then the reaction mixture was stirred and refluxed for 48 h. At the end of this period, the mixture was diluted with H_2_O (10 mL) and extracted with dichloromethane (3 × 10 mL). The organic layer was washed with H_2_O (15 mL), dried over anhydrous sodium sulfate, and the volatile fraction was evaporated under reduced pressure. The residue was purified by silica gel column chromatography (dichloromethane/hexane 1:1, *v*/*v*).

2-(4-Fluorophenyl)-3-(1*H*-indol-1-yl)-1,2-epoxypropane (**7a**). Yellow oil (40% yield); Rf = 0.68 (dichloromethane); IR (NaCl): ν 1620, 1366, 1289 cm^−1^; ^1^H NMR (DMSO-*d_6_*): δ 7.59 (d, 1H, *J* = 7.6 Hz, H_4″_), 7.49–7.52 (m, 1H, H_7″_), 7.44–7.49 (m, 2H, H_2′_, H_6′_), 7.27 (d, 1H, *J* = 3.1 Hz, H_2″_), 7.10–7.17 (m, 3H, H_3′_, H_5′,_ H_6″_), 6.98–7.04 (m, 1H, H_5″_), 6.40 (d, 1H, *J* = 3.1 Hz, H_3″_), 5.07 (d, 1H, *J* = 15.7 Hz, H_3_), 4.57 (d, 1H, *J* = 15.7 Hz, H_3_), 2.81 (d, 1H, *J* = 4.9 Hz, H_1_), 2.93 (d, 1H, *J* = 4.9 Hz, H_1_).

2-(4-Chlorophenyl)-3-(1*H*-indol-1-yl)-1,2-epoxypropane (**7b**). Colorless oil (75% yield); Rf = 0.68 (dichloromethane); IR (NaCl): ν 1598, 1315, 1270 cm^−1^; ^1^H NMR (DMSO-*d_6_*): δ 7.58–7.61 (m, 1H, H_4″_), 7.48–7.51 (m, 1H, H_7″_), 7.43–7.46 (m, 2H, H_3′_, H_5′_), 7.36 (d, 2H, *J* = 8.6 Hz, H_2′_, H_6′_), 7.26 (d, 1H, *J* = 3.1 Hz, H_2″_), 7.10–7.16 (m, 1H, H_6″_), 6.97–7.03 (m, 1H, H_5″_), 6.38 (d, 1H, *J* = 3.1 Hz, H_3″_), 5.09 (d, 1H, *J* = 15.6 Hz, H_3_), 4.54 (d, 1H, *J* = 15.6 Hz, H_3_), 2.95 (d, 1H, *J* = 4.9 Hz, H_1_), 2.80 (d, 1H, *J* = 4.9 Hz, H_1_).

2-(4-Bromophenyl)-3-(1*H*-indol-1-yl)-1,2-epoxypropane (**7c**). Colorless oil (37% yield); Rf = 0.62 (dichloromethane); IR (NaCl): ν 1600, 1320, 1266 cm^−1^; ^1^H NMR (DMSO-*d_6_*): δ 7.59–7.62 (m, 1H, H_4″_), 7.51 (d, 2H, *J* = 8.5 Hz, H_3′_, H_5′_), 7.42–7.44 (m, 1H, H_7″_), 7.40 (d, 2H, *J* = 8.5 Hz, H_2′_, H_6′_), 7.29 (d, 1H, *J* = 3.1 Hz, H_2″_), 7.14 (dd, 1H, *J* = 8.2 Hz, *J* = 7.0 Hz, H_6″_), 6.97–7.05 (m, 1H, H_5″_), 6.39 (d, 1H, *J* = 3.1 Hz, H_3″_), 5.11 (d, 1H, *J* = 15.6 Hz, H_3_), 4.55 (d, 1H, *J* = 15.6 Hz, H_3_), 2.96 (d, 1H, *J* = 4.9 Hz, H_1_), 2.81 (d, 1H, *J* = 4.9 Hz, H_1_).

2-(4-Trifluoromethylphenyl)-3-(1*H*-indol-1-yl)-1,2-epoxypropane (**7d**). Yellow oil (62% yield); IR (NaCl): ν 1592, 1312, 1271 cm^−1^; ^1^H NMR (DMSO-*d_6_*): δ 7.68 (s, 4H, H_2′_, H_3′,_ H_5′_, H_6′_), 7.64 (d, 1H, *J* = 8.6 Hz, H_4″_), 7.51 (d, 1H, *J* = 8.6 Hz, H_7″_), 7.29 (d, 1H, *J* = 4.0 Hz, H_2″_), 7.15 (dd, 1H, *J = J =* 8.6 Hz, H_5″_), 7.02 (dd, 1H, *J = J =* 8.6 Hz, H_6″_), 6.40 (d, 1H, *J* = 4.0 Hz, H_3″_), 5.20 (d, 1H, *J* = 15.3 Hz, H_3_), 4.59 (d, 1H, *J* = 15.3 Hz, H_3_), 3.03 (d, 1H, *J* = 4.9 Hz, H_1_), 2.85 (d, 1H, *J* = 4.9 Hz, H_1_).

2-(2,4-Difluorophenyl)-3-(1*H*-indol-1-yl)-1,2-epoxypropane (**7e**). Yellow oil (59% yield); Rf = 0.59 (dichloromethane); IR (NaCl): ν 1589, 1369, 1290 cm^−1^; ^1^H NMR (DMSO-*d_6_*): δ 7.56 (d, 1H, *J* = 7.6 Hz, H_4″_), 7.52 (d, 1H, *J* = 7.6 Hz, H_7″_), 7.43 (d, 1H, *J* = 7.9 Hz, H_6′_), 7.18 (d, 1H, *J* = 3.1 Hz, H_2″_), 7.11–7.15 (m, 1H, H_3′_), 7.07–7.10 (m, 1H, H_5′_), 6.98–7.04 (m, 1H, H_6″_), 6.91–6.98 (m, 1H, H_5″_), 6.40 (d, 1H, *J* = 3.1 Hz, H_3″_), 4.81 (d, 1H, *J* = 15.5 Hz, H_3_), 4.53 (d, 1H, *J* = 15.5 Hz, H_3_), 2.98 (d, 1H, *J* = 4.9 Hz, H_1_), 2.91 (d, 1H, *J* = 4.9 Hz, H_1_).

2-(2,4-Dichlorophenyl)-3-(1*H*-indol-1-yl)-1,2-epoxypropane (**7f**). Green oil (61% yield); Rf = 0.64 (dichloromethane); IR (NaCl): ν 1612, 1371, 1314 cm^−1^; ^1^H NMR (DMSO-*d_6_*): δ 7.68 (d, 1H, *J* = 1.8 Hz, H_3′_), 7.52 (d, 1H, *J* = 7.3 Hz, H_4″_), 7.40 (d, 1H, *J* = 7.9 Hz, H_7″_), 7.17 (d, 1H, *J* = 3.1 Hz, H_2″_), 7.09–7.15 (m, 1H, H_6″_), 7.07–7.11 (m, 1H, H_5′_), 7.01–7.04 (m, 1H, H_6′_), 6.98–7.04 (m, 1H, H_5″_), 6.41 (d, 1H, *J* = 3.1 Hz, H_3″_), 4.90 (d, 1H, *J* = 15.6 Hz, H_3_), 4.51 (d, 1H, *J* = 15.6 Hz, H_3_), 3.01 (d, 1H, *J* = 4.6 Hz, H_1_), 2.90 (d, 1H, *J* = 4.6 Hz, H_1_).

#### 3.2.7. General Procedure for the Synthesis of Imidazole Derivatives **8a**–**e**

Potassium carbonate (0.25 g, 1.83 mmol) and *1H*-imidazole (0.12 g, 1.82 mmol) were added to a solution of the corresponding oxirane **7a**–**e** (0.64 mmol) in dimethylformamide (20 mL). Then the reaction mixture was stirred and refluxed for 7 h. At the end of this period, the mixture was diluted with H_2_O (20 mL) and extracted with EtOAc (3 × 40 mL). The organic layer was washed with brine (40 mL), dried over anhydrous sodium sulfate, and the volatile fraction was evaporated under reduced pressure. The residue was purified by silica gel column chromatography (dichloromethane/ethanol 19:1, *v*/*v*).

2-(4-Fluorophenyl)-1-(1*H*-imidazol-1-yl)-3-(1*H*-indol-1-yl)propan-2-ol (**8a**). White powder (50% yield); Rf = 0.13 (dichloromethane /EtOH: 19/1); mp: 166–167 °C; IR (KBr): ν 3438 (w, OH), 1607, 1329 cm^−1^; ^1^H NMR (DMSO-*d_6_*): δ 7.44–7.49 (m, 2H, H_4‴,_ H_7‴_), 7.35 (s, 1H, H_2″_), 7.15 (d, 1H, *J* = 3.1 Hz, H_2‴_), 6.87 (s, 1H, H_4″_), 6.72 (s, 1H, H_5″_), 6.97–7.09 (m, 6H, H_5‴_, H_6‴_, H_2′_, H_3′_, H_5′_, H_6′_), 6.37 (d, 1H, *J* = 3.1 Hz, H_3‴_), 5.94 (s, 1H, OH), 4.63 (d, 1H, *J* = 14.4 Hz, H_3_), 4.57 (d, 1H, *J* = 14.1 Hz, H_1_), 4.43 (d, 1H, *J* = 14.1 Hz, H_1_), 4.29 (d, 1H, *J* = 14.4 Hz, H_3_); MS *m*/*z*: 335 (M^+^), 240, 130 (100), 81. Anal. Calcd. for C_20_H_18_FN_3_O (335.37): C: 71.62; H: 5.37; N: 12.52; found: C: 71.61; H: 5.36; N: 12.50%.

2-(4-Chlorophenyl)-1-(1*H*-imidazol-1-yl)-3-(1*H*-indol-1-yl)propan-2-ol (**8b**). White powder (60% yield); Rf = 0.15 (dichloromethane /EtOH: 19/1); mp: 201–202 °C; IR (KBr): ν 3120 (w, OH), 1609, 1317 cm^−1^; ^1^H NMR (DMSO-*d_6_*): δ 7.51 (d, 2H, *J* = 8.5 Hz, H_3′_, H_5′_), 7.49 (d, 1H, *J* = 7.5 Hz, H_4‴_), 7.48 (d, 1H, *J* = 8.0 Hz, H_7‴_), 7.35 (s, 1H, H_2″_), 7.30 (d, 2H, *J* = 8.5 Hz, H_2′_, H_6′_), 7.16 (d, 1H, *J* = 3.1 Hz, H_2‴_), 7.08 (dd, 1H, *J* = 8.0 Hz, *J* = 7.0 Hz, H_6‴_), 6.98 (dd, 1H, *J* = 7.5 Hz, *J* = 7.0 Hz, H_5‴_), 6.89 (s, 1H, H_4″_), 6.72 (s, 1H, H_5″_), 6.37 (d, 1H, *J* = 3.1 Hz, H_3‴_), 5.99 (s, 1H, OH), 4.63 (d, 1H, *J* = 14.3 Hz, H_3_), 4.59 (d, 1H, *J* = 14.4 Hz, H_1_), 4.44 (d, 1H, *J* = 14.4 Hz, H_1_), 4.29 (d, 1H, *J* = 14.3 Hz, H_3_); MS *m*/*z*: 351, 353 (M^+^), 240, 130 (100), 81. Anal. Calcd. for C_20_H_18_ClN_3_O (351.83): C: 68.27; H: 5.12; N: 11.94; found: C: 68.26; H: 5.11; N: 11.92%.

2-(4-Bromophenyl)-1-(1*H*-imidazol-1-yl)-3-(1*H*-indol-1-yl)propan-2-ol (**8c**). White powder (21% yield); Rf = 0.13 (dichloromethane /EtOH: 19/1); mp: 134–135 °C; IR (KBr): ν 3054 (w, OH), 1602, 1313 cm^−1^; ^1^H NMR (DMSO-*d_6_*): δ 7.62 (s, 1H, H_2″_), 7.45–7.51 (m, 5H, H_2′,_ H_3′,_ H_5′,_ H_6′,_ H_4‴_), 7.45–7.48 (m, 1H, H_7‴_), 7.19 (d, 1H, *J* = 3.1 Hz, H_2‴_), 7.06–7.12 (m, 1H, H_6‴_), 6.99 (dd, 1H, *J = J =* 7.0 Hz, H_5‴_), 6.96 (s, 1H, H_4″_), 6.87 (s, 1H, H_5″_), 6.40 (d, 1H, *J* = 3.1 Hz, H_3‴_), 6.09 (s, 1H, OH), 4.70 (d, 1H, *J* = 14.3 Hz, H_3_), 4.60 (d, 1H, *J* = 14.4 Hz, H_1_), 4.48 (d, 1H, *J* = 14.4 Hz, H_1_), 4.32 (d, 1H, *J* = 14.3 Hz, H_3_); MS *m*/*z*: 395, 397 (M^+^), 240, 130 (100), 81. Anal. Calcd. for C_20_H_18_BrN_3_O (396.28): C: 60.61; H: 4.54; N: 10.60; found: C: 60.58; H: 4.55; N: 10.58%.

2-(2,4-Difluorophenyl)-1-(1*H*-imidazol-1-yl)-3-(1*H*-indol-1-yl)propan-2-ol (**8d**). White powder (50% yield); Rf = 0.17 (dichloromethane /EtOH: 19/1); mp: 166–168 °C; IR (KBr): ν 3424 (w, OH), 1616, 1317 cm^−1^; ^1^H NMR (DMSO-*d_6_*): δ 7.49 (d, 1H, *J* = 7.6 Hz, H_4‴_), 7.42 (s, 1H, H_2″_), 7.39–7.42 (m, 1H, H_7‴_), 7.25–7.28 (m, 1H, H_6′_), 7.20–7.21 (m, 2H, H_2‴_, H_4″_), 7.07–7.13 (m, 1H, H_6‴_), 6.98–7.01 (m, 1H, H_5′_), 6.89–6.95 (m, 1H, H_3′_), 6.83–6.89 (m, 1H, H_5‴_), 6.73 (s, 1H, H_5″_), 6.39–6.40 (m, 1H, H_3‴_), 6.24 (s, 1H, OH), 4.68 (d, 1H, *J* = 14.4 Hz, H_3_), 4.68 (d, 1H, *J* = 14.3 Hz, H_1_), 4.49 (d, 1H, *J* = 14.3 Hz, H_1_), 4.30 (d, 1H, *J* = 14.4 Hz, H_3_); MS *m*/*z*: 353 (M^+^), 240, 130 (100), 81. Anal. Calcd. for C_20_H_17_F_2_N_3_O (353.37): C: 67.97; H: 4.81; N: 11.89; found: C: 67.95; H: 4.79; N: 11.86%.

2-(2,4-Dichlorophenyl)-1-(1*H*-imidazol-1-yl)-3-(1*H*-indol-1-yl)propan-2-ol (**8e**). White powder (70% yield); Rf = 0.16 (dichloromethane /EtOH: 19/1); mp: 212–213 °C; IR (KBr): ν 3408 (w, OH), 1609, 1329 cm^−1^; ^1^H NMR (DMSO-*d_6_*): δ 7.98 (s, 1H, H_2″_), 7.57–7.61 (m, 1H, H_3′_), 7.44–7.48 (m, 1H, H_4‴_), 7.36 (d, 1H, *J* = 8.5 Hz, H_7‴_), 7.23 (d, 1H, *J* = 3.1 Hz, H_2‴_), 7.16–7.19 (m, 2H, H_4′_, H_5′_), 7.05–7.11 (m, 1H, H_6‴_), 6.91 (s, 1H, H_4″_), 6.87–6.91 (m, 1H, H_5‴_), 6.68 (s, 1H, H_5″_), 6.35 (d, 1H, *J* = 3.1 Hz, H_3‴_), 6.01 (s, 1H, OH), 5.15 (d, 1H, *J* = 14.4 Hz, H_3_), 4.96 (d, 1H, *J* = 14.3 Hz, H_1_), 4.75 (d, 1H, *J* = 14.3 Hz, H_1_), 4.47 (d, 1H, *J* = 14.4 Hz, H_3_); MS *m*/*z*: 385, 387, 389 (M^+^), 240, 130 (100), 81. Anal. Calcd. for C_20_H_17_Cl_2_N_3_O (386.27): C: 62.18; H: 4.40; N: 10.87; found: C: 62.20; H: 4.43; N: 10.88%.

#### 3.2.8. General Procedure for the Synthesis of Triazole Derivatives **8f** and **8g**

Potassium carbonate (0.25 g, 1.83 mmol) and 1,2,4-1*H*-triazole (0.13 g, 1.82 mmol) were added to a solution of the corresponding oxirane **7d**,**f** (0.64 mmol) in dimethylformamide (20 mL). Then the reaction mixture was stirred and refluxed for 7 h. At the end of this period, the mixture was diluted with H_2_O (20 mL) and extracted with EtOAc (40 mL). The organic layer was washed with brine (40 mL), dried over anhydrous sodium sulfate, and the volatile fraction was evaporated under reduced pressure. The residue was purified by silica gel column chromatography (dichloromethane/ethanol 19:1, *v*/*v*).

1-(1*H*-Indol-1-yl)-3-(1,2,4-1*H*-triazol-1-yl)-2-(4-trifluoromethyl-phenyl)propan-2-ol (**8f**). White powder (86% yield); mp: 165–168 °C; IR (KBr): ν 3420 (w, OH), 1613, 1307 cm^−1^; ^1^H NMR (DMSO-*d_6_*): δ 8.32 (s, 1H, H_3″_), 7.88 (s, 1H, H_5″_), 7.68 (d, 2H, *J* = 8.0 Hz, H_3″_, H_5‴_), 7.58 (d, 2H, *J* = 8.0 Hz, H_2″_, H_6‴_), 7.48 (d, 1H, *J* = 7.6 Hz, H_4′_), 7.40 (d, 1H, *J* = 7.6 Hz, H_7′_), 7.29 (d, 1H, *J* = 2.8 Hz, H_2′_), 7.03 (dd, 1H, *J* = 7.6 Hz, H_6′_), 6.96 (dd, 1H, *J* = 7.6 Hz, H_5′_), 6.41 (d, 1H, *J* = 2.8 Hz, H_3′_), 6.16 (s, 1H, OH), 4.92 (d, 1H, *J* = 14.9 Hz, H_1_), 4.64 (d, 1H, *J* = 14.9 Hz, H_1_), 4.61 (s, 2H, H_3_); MS *m*/*z*: 386 (M^+^), 241, 130 (100), 82. Anal. Calcd. for C_20_H_17_F_3_N_4_O (386.37): C: 62.17; H: 4.40; N: 14.49; found: C: 62.12; H: 4.41; N: 14.47%.

2-(2,4-Dichlorophenyl)-3-(1*H*-indol-1-yl)-1-(1*H*-1,2,4-triazol-1-yl)-propan-2-ol (**8g**). White powder (45% yield); Rf = 0.11 (AcOEt/hexane: 1/1); mp: 161–162 °C; IR (KBr): ν 3423 (w, OH), 1585, 1273 cm^−1^; ^1^H NMR (DMSO-*d_6_*): δ 8.32 (s, 1H, H_3‴_), 7.79 (s, 1H, H_5‴_), 7.63 (d, 1H, *J* = 2.1 Hz, H_3′_), 7.50 (d, 1H, *J* = 8.2 Hz, H_6′_), 7.50 (d, 1H, *J* = 7.9 Hz, H_4″_), 7.42 (d, 1H, *J* = 7.9 Hz, H_7″_), 7.23 (dd, 1H, *J* = 8.2 Hz, *J* = 2.1 Hz, H_5′_), 7.21–7.23 (m, 1H, H_2″_), 7.13 (dd, 1H, *J = J =* 7.9 Hz, H_6″_), 7.01 (dd, 1H, *J = J =* 7.9 Hz, H_5″_), 6.40 (d, 1H, *J* = 2.7 Hz, H_3″_), 6.36 (s, 1H, OH), 5.31 (d, 1H, *J* = 14.3 Hz, H_3_), 4.88 (d, 1H, *J* = 15.0 Hz, H_1_), 4.78 (d, 1H, *J* = 15.0 Hz, H_1_), 4.55 (d, 1H, *J* = 14.3 Hz, H_3_); ^13^C NMR (DMSO-*d_6_*): 50.8, 53.4, 76.7, 101.2, 110.1, 118.2, 120.2, 121.1, 127.0, 127.6, 129.6, 129.9, 131.2, 131.3, 133.1, 136.9, 137.0, 145.4, 151.2; MS *m*/*z*: 386, 388, 390 (M^+^), 241, 130 (100), 82. Anal. Calcd. for C_19_H_16_Cl_2_N_4_O (387.26): C: 58.92; H: 4.13; N: 14.46; found: C: 58.89; H: 4.11; N: 14.45%.

### 3.3. Synthesis of Compounds ***8g*** and ***11a***–***k*** (Route 2)

#### 3.3.1. Synthesis of 2-(1*H*-Imidazol-1-yl)-1-(4-trifluoromethylphenyl)ethanone (**9f**)

To a solution of 2-bromo-4′-trifluoromethylacetophenone (2.99 g, 11.19 mmol) in acetonitrile (40 mL) was added 1*H*-imidazole (1.52 g, 22.37 mmol) and K_2_CO_3_ (3.09 g, 22.37 mmol). The reaction mixture was irradiated for 50 min in a microwave oven, programmed to obtain reflux with a maximum power output of 50 W. After cooling, the mixture was filtered and evaporated under reduce pressure. The residue was diluted with H_2_O (40 mL) and extracted with dichloromethane (3 x 40 mL), then the organic layer was washed with brine (40 mL), dried over anhydrous sodium sulfate, and the volatile fraction was evaporated under reduced pressure. The residue was purified by silica gel column chromatography (ethanol/dichloromethane 1:10, *v*/*v*). 2-(1*H*-Imidazol-1-yl)-1-(4-trifluoromethylphenyl)ethanone (**9f**) was obtained (80% yield): White powder; Rf = 0.80 (EtOH/dichloromethane: 1/10); mp: 115–116 °C; IR (KBr): ν 1702 (s, C=O), 1603, 1228 cm^−1^; ^1^H NMR (DMSO-*d_6_*): δ 8.26 (d, 2H, *J* = 8.2 Hz, H_3′_, H_5′_); 8.02 (d, 2H, *J* = 8.2 Hz, H_2′_, H_6′_); 7.63 (s, 1H, H_2″_), 7.17 (s, 1H, H_4″_), 6.97 (s, 1H, H_5″_), 5.84 (s, 2H, H_2_). ^13^C NMR (DMSO-*d_6_*): δ 53.3, 121.2, 124.2 (q, *^1^JCF =* 287.1 Hz), 126.3 (q, 2C, *^3^JCF =* 3.8 Hz), 129.2, 129.4, 133.4 (q, *^2^JCF =* 32.0 Hz), 137.4, 138.1, 193.6.

#### 3.3.2. Synthesis of 3-(1*H*-imidazol-1-yl)-2-(4-trifluoromethylphenyl)-1,2-epoxypropane (**10f**)

Trimethylsulfoxonium iodide (4.59 g, 20.85 mmol) and sodium hydroxide (4.17 g, 104.26 mmol, 20% in aqueous solution) were added to a solution of 2-(1*H*-imidazol-1-yl)-1-(4-trifluoromethylphenyl)ethanone (**9f**) (2.65 g, 10.43 mmol) in dichloromethane (50 mL). Then the reaction mixture was stirred and refluxed for 72 h. At the end of this period, the mixture was diluted with H_2_O (50 mL) and extracted with dichloromethane (3 × 50 mL). The organic layer was washed with H_2_O (50 mL), dried over anhydrous sodium sulfate, and the volatile fraction was evaporated under reduced pressure. The residue was purified by silica gel column chromatography (dichloromethane/ethanol 10:1, *v*/*v*). 3-(1*H*-Imidazol-1-yl)-2-(4-trifluoromethylphenyl)-1,2-epoxypropane (**10f**) was obtained (53% yield): Red oil; Rf = 0.91 (EtOH/dichloromethane: 1/10); IR (NaCl): ν 1541, 1328, 1073 cm^−1^; ^1^H NMR (DMSO-*d_6_*): δ 7.75 (d, 2H, *J* = 7.9 Hz, H_3′_, H_5′_); 7.64 (d, 2H, *J* = 7.9 Hz, H_2′_, H_6′_); 7.56 (s, 1H, H_2″_); 7.10 (s, 1H, H_4″_); 6.85 (s, 1H, H_5″_); 5.04 (d, 1H, *J* = 15.0 Hz, H_3_); 4.38 (d, 1H, *J* = 15.0 Hz, H_3_); 3.10 (d, 1H, *J* = 4.9 Hz, H_1_); 2.90 (d, 1H, *J* = 4.9 Hz, H_1_). ^13^C NMR (DMSO-*d_6_*): δ 49.6, 53.8, 59.1, 118.2, 124.1 (q, *^1^JCF =* 267.9 Hz), 125.3 (q, 2C, *^3^JCF =* 3.8 Hz), 126.7, 128.3, 128.5 (q, *^2^JCF =* 31.9 Hz), 138.1, 141.6.

#### 3.3.3. General Procedure for the *N-*Alkylation of Indole Derivatives **8g** and **11a**–**k**

Sodium hydride (0.06 g, 2.61 mmol) was dissolved in DMSO (20 mL) and the corresponding indole derivative (2.61 mmol) was added portionwise, then the mixture was stirred at rt for 1 h under argon. After this period, the corresponding oxirane derivative **10a**–**f** (2.61 mmol) in DMSO (10 mL) was added and the mixture was stirred for 12 h. At the end of this period, the mixture was diluted with H_2_O (20 mL) and extracted with ethyl acetate (3 × 40 mL). The organic layer was washed with brine (40 mL), dried over anhydrous sodium sulfate, and the volatile fraction was evaporated under reduced pressure. The residue was purified by silica gel column chromatography (EtOAc/hexane 1:1, *v*/*v*).

2-(4-Fluorophenyl)-3-(1*H*-indol-1-yl)-1-(1*H*-1,2,4-triazol-1-yl)propan-2-ol (**11a**). White powder (57% yield); Rf = 0.08 (EtOAc/hexane: 1/1); mp: 190–191 °C; IR (KBr): ν 3408 (w, OH), 1602, 1217 cm^−1^; ^1^H NMR (DMSO-*d_6_*): δ 8.27 (s, 1H, H_3‴_), 7.90 (s, 1H, H_5‴_), 7.44–7.49 (m, 3H, H_2′_, H_6′_, H_4″_), 7.40 (d, 1H, *J* = 7.0 Hz, H_4″_), 7.27 (d, 1H, *J* = 3.1 Hz, H_2″_), 7.05 (dd, 1H, *J = J =* 7.0 Hz, H_6″_), 7.04 (dd, 2H, *J = J =* 8.9 Hz, H_3′_, H_5′_), 6.97 (dd, 1H, *J = J =* 7.0 Hz, H_5″_), 6.39 (d, 1H, *J* = 3.1 Hz, H_3″_), 6.00 (s, 1H, OH), 4.82 (d, 1H, *J* = 14.3 Hz, H_3_), 4.60 (d, 1H, *J* = 15.3 Hz, H_1_), 4.54 (d, 1H, *J* = 15.3 Hz, H_1_), 4.53 (d, 1H, *J* = 14.3 Hz, H_3_); ^13^C NMR (DMSO-*d_6_*): δ 53.6, 56.1, 75.9, 100.8, 110.3, 114.3 (d, 2C, *^2^JCF =* 21.5 Hz), 118.7, 120.0, 120.7, 127.5, 129.8, 128.0 (d, 2C, *^3^JCF =* 8.1 Hz), 137.0, 137.5 (d, *^4^JCF =* 3.3 Hz), 145.0, 150.6, 161.3 (d, *^1^JCF =* 242.7 Hz); MS *m*/*z* 336 (M^+^), 241, 130 (100), 82. Anal. Calcd. for C_19_H_17_FN_4_O (336.36): C: 67.84; H: 5.05; N: 16.65; found: C: 67.87; H: 5.03; N: 16.61%.

2-(2,4-Dichlorophenyl)-3-(1*H*-indol-1-yl)-1-(1*H*-1,2,4-triazol-1-yl)propan-2-ol (**8g**). White powder (38% yield); all physicochemical and spectral data were similar to **8g** obtained by route 1.

2-(4-Chlorophenyl)-3-(1*H*-indol-1-yl)-1-(1*H*-1,2,4-triazol-1-yl)propan-2-ol (**11b**). White powder (89% yield); Rf = 0.77 (EtOH/dichloromethane: 1/10); mp: 188–189 °C; IR (KBr): ν 3408 (w, OH), 1596, 1269 cm^−1^; ^1^H NMR (DMSO-*d_6_*): δ 8.28 (s, 1H, H_3‴_), 7.89 (s, 1H, H_5‴_), 7.49 (d, 1H, *J* = 7.0 Hz, H_4″_), 7.47 (d, 2H, *J* = 8.9 Hz, H_2′,_ H_6′_), 7.44 (d, 1H, *J* = 7.0 Hz, H_7″_), 7.28 (d, 2H, *J* = 8.9 Hz, H_3′_, H_5′_), 7.27 (d, 1H, *J* = 2.7 Hz, H_2″_), 7.06 (dd, 1H, *J = J =* 7.0 Hz, H_6″_), 6.98 (dd, 1H, *J = J =* 7.0 Hz, H_5″_), 6.40 (d, 1H, *J* = 2.7 Hz, H_3″_), 6.05 (s, 1H, OH), 4.84 (d, 1H, *J* = 14.0 Hz, H_3_), 4.62 (d, 1H, *J* = 15.0 Hz, H_1_), 4.60 (d, 1H, *J* = 14.0 Hz, H_3_), 4.54 (d, 1H, *J* = 15.0 Hz, H_1_); ^13^C NMR (DMSO-*d_6_*): δ 53.5, 56.0, 76.0, 100.9, 110.4, 118.7, 119.9, 120.7, 120.8, 125.1, 127.5 (2C), 127.9 (2C), 129.8, 137.0, 140.4, 145.0, 150.6; MS *m*/*z*: 352 (M^+^), 354 (M+2), 241, 130 (100), 82. Anal. Calcd. for C_19_H_17_ClN_4_O (352.82): C: 64.68; H: 4.82; N: 15.87; found: C: 64.64; H: 4.79; N: 15.89%.

2-(4-Bromophenyl)-3-(1*H*-indol-1-yl)-1-(1*H*-1,2,4-triazol-1-yl)propan-2-ol (**11c**). White powder (45% yield); Rf = 0.09 (EtOAc/hexane: 1/1); mp: 186–187 °C; IR (KBr): ν 3406 (w, OH), 1587, 1271 cm^−1^; ^1^H NMR (DMSO-*d_6_*): δ 8.27 (s, 1H, H_3‴_), 7.88 (s, 1H, H_5‴_), 7.48 (d, 1H, *J* = 7.0 Hz, H_4″_), 7.44 (d, 1H, *J* = 7.0 Hz, H_7″_), 7.41 (s, 4H, H_2′_, H_3′_, H_5′_, H_6′_), 7.26 (d, 1H, *J* = 3.1 Hz, H_2″_), 7.06 (dd, 1H, *J = J =* 7.0 Hz, H_6″_), 6.98 (dd, 1H, *J = J =* 7.0 Hz, H_5″_), 6.40 (d, 1H, *J* = 3.1 Hz, H_3″_), 6.04 (s, 1H, OH), 4.84 (d, 1H, *J* = 14.3 Hz, H_3_), 4.61 (d, 1H, *J* = 15.0 Hz, H_1_), 4.57 (d, 1H, *J* = 14.3 Hz, H_3_), 4.54 (d, 1H, *J* = 15.0 Hz, H_1_); ^13^C NMR (DMSO-*d_6_*): δ 53.5, 55.9, 76.0, 100.9, 110.4, 118.7, 120.0, 120.5, 120.8, 127.5, 128.3 (2C), 129.8, 130.5 (2C), 137.0, 140.9, 145.0, 150.6; MS *m*/*z*: 396, 398 (M^+^), 241, 130 (100), 82. Anal. Calcd. for C_19_H_17_BrN_4_O (397.27): C: 57.44; H: 4.28; N: 14.10; found: C: 57.40; H: 4.25; N: 14.08%.

2-(2,4-Difluorophenyl)-3-(1*H*-indol-1-yl)-1-(1*H*-1,2,4-triazol-1-yl)-propan-2-ol (**11d**). White powder (41% yield); Rf = 0.08 (EtOAc/hexane: 1/1); mp: 119–120 °C; IR (KBr): ν 3206 (w, OH), 1615, 1313 cm^−1^; ^1^H NMR (DMSO-*d_6_*): δ 8.34 (s, 1H, H_3‴_), 7.82 (s, 1H, H_5‴_), 7.49 (d, 1H, *J* = 7.6 Hz, H_4″_), 7.39 (d, 1H, *J* = 7.6 Hz, H_7″_), 7.25 (d, 1H, *J* = 3.1 Hz, H_2″_), 7.17–7.29 (m, 2H, H_3′_, H_5′_), 7.10 (dd, 1H, *J = J =* 7.6 Hz, H_6″_), 6.99 (dd, 1H, *J = J =* 7.6 Hz, H_5″_), 6.86 (td, 1H, *J* = 8.6Hz, *J* = 2.4 Hz, H_2′_), 6.40 (d, 1H, *J* = 3.1 Hz, H_3″_), 6.29 (s, 1H, OH), 4.90 (d, 1H, *J* = 14.3 Hz, H_3_), 4.68 (d, 1H, *J* = 15.0 Hz, H_1_), 4.57 (d, 1H, *J* = 15.0 Hz, H_1_), 4.56 (d, 1H, *J* = 14.3 Hz, H_3_); ^13^C NMR (DMSO-*d_6_*): δ 52.1, 54.8, 75.2 (d, *^3^JCF =* 5.2 Hz), 100.9, 103.9 (dd, *^2^JCF = ^2^JCF =* 26.2 Hz), 111.0 (dd, *^2^JCF =* 20.5 Hz, *^4^JCF =* 2.9 Hz), 111.3, 118.7, 120.0, 120.8, 124.0 (dd, *^2^JCF =* 13.4 Hz, *^4^JCF =* 3.8 Hz), 125.1, 129.6, 130.1 (dd, *^3^JCF =* 10.0 Hz, *^3^JCF =* 6.7 Hz), 136.9, 145.0, 150.7, 158.9 (dd, *^1^JCF =* 247.0 Hz, *^3^JCF =* 12.4 Hz), 162.5 (dd, *^1^JCF =* 246.1 Hz, *^3^JCF =* 12.9 Hz); MS *m*/*z*: 354 (M^+^), 241, 130 (100), 82. Anal. Calcd. for C_19_H_16_F_2_N_4_O (354.35): C: 64.40; H: 4.52; N: 15.80; found: C: 64.43; H: 4.50; N: 15.84%.

1-(1*H*-Imidazol-1-yl)-3-(1*H*-indol-1-yl)-2-(4-trifluoromethylphenyl)propan-2-ol (**11e**). Orange powder (9% yield); Rf = 0.61 (EtOAc/hexane: 1/1); mp: 246–247 °C; IR (KBr): ν 3421 (w, OH), 1610, 1328 cm^−1^; ^1^H NMR (DMSO-*d_6_*): δ 7.73 (d, 2H, *J* = 8.2 Hz, H_3‴_, H_5‴_), 7.60 (d, 2H, *J* = 8.2 Hz, H_2‴_, H_6‴_), 7.48 (d, 1H, *J* = 7.9 Hz, H_4″_), 7.45 (d, 1H, *J* = 7.9 Hz, H_7″_), 7.38 (s, 1H, H_2′_), 7.18 (d, 1H, *J* = 3.1 Hz, H_2″_), 7.07 (dd, 1H, *J = J =* 7.9 Hz, H_6″_), 6.94 (dd, 1H, *J = J =* 7.9 Hz, H_5″_), 6.91 (s, 1H, H_5′_), 6.72 (s, 1H, H_4′_), 6.38 (d, 1H, *J* = 3.1 Hz, H_3″_), 6.16 (s, 1H, OH), 4.72 (d, 1H, *J* = 14.3 Hz, H_3_), 4.63 (d, 1H, *J* = 14.7 Hz, H_1_), 4.48 (d, 1H, *J* = 14.7 Hz, H_1_), 4.35 (d, 1H, *J* = 14.3 Hz, H_3_); ^13^C NMR (DMSO-*d_6_*): δ 53.6, 56.9, 76.8, 100.5, 110.4, 117.4, 119.1, 119.9, 120.6, 124.2 (q, 2C, *^3^JCF =* 5.7 Hz), 124.8 (q, *^1^JCF =* 263.4 Hz), 127.2 (2C), 127.4, 128.3, 128.9 (q, *^2^JCF =* 26.7 Hz), 130.6, 138.1, 138.6, 139.9; MS *m*/*z*: 385 (M^+^), 240, 130 (100), 81. Anal. Calcd. for C_21_H_18_F_3_N_3_O (385.38): C: 65.44; H: 4.67; N: 10.90; found: C: 65.41; H: 4.64; N: 10.87%.

2-(2,4-Difluorophenyl)-3-(2-methyl-1*H*-indol-1-yl)-1-(1*H*-1,2,4-triazol-1-yl)propan-2-ol (**11f**). Orange powder (34% yield); Rf = 0.17 (EtOAc/hexane: 1/10); mp: 125–126 °C; IR (KBr): ν 3113 (w, OH), 1612, 1270 cm^−1^; ^1^H NMR (DMSO-*d_6_*): δ 8.38 (s, 1H, H_3‴_), 7.82 (s, 1H, H_5‴_), 7.27–7.42 (m, 2H, H_3′_, H_5′_), 7.41 (d, 1H, *J* = 7.0 Hz, H_4″_), 7.25 (d, 1H, *J* = 7.0 Hz, H_7″_), 6.96–7.04 (m, 2H, H_5″_, H_6″_), 6.93 (td, 1H, *J* = 8.2 Hz, *J* = 2.4 Hz, H_6′_), 6.22 (s, 1H, H_3″_), 6.09 (s, 1H, OH), 4.96 (d, 1H, *J* = 14.4 Hz, H_3_), 4.61 (d, 1H, *J* = 15.3 Hz, H_1_), 4.59 (d, 1H, *J* = 14.4 Hz, H_3_), 4.46 (d, 1H, *J* = 15.3 Hz, H_1_), 2.22 (s, 3H, CH_3_); ^13^C NMR (DMSO-*d_6_*): δ 13.0, 52.1, 54.8, 75.2 (d, *^3^JCF =* 5.2 Hz), 100.9, 103.9 (dd, *^2^JCF = ^2^JCF =* 26.2 Hz), 111.0 (dd, *^2^JCF =* 20.5 Hz, *^4^JCF =* 2.9Hz), 111.3, 118.7, 120.0, 120.8, 124.0 (dd, *^2^JCF =* 3.4 Hz, *^4^JCF =* 3.8 Hz), 125.1, 129.6, 130.1 (dd, *^3^JCF =* 10.0 Hz, *^3^JCF =* 6.7 Hz), 136.9, 145.0, 150.7, 158.9 (dd, *^1^JCF =* 247.0 Hz, *^3^JCF =* 12.4 Hz), 162.5 (dd, *^1^JCF =* 246.1 Hz, *^3^JCF =* 12.9 Hz); MS *m*/*z*: 368 (M^+^), 255, 144 (100), 82. Anal. Calcd. for C_20_H_18_F_2_N_4_O (368.38): C: 65.20; H: 4.89; N: 15.20; found: C: 65.18; H: 4.91; N: 15.17%.

2-(2,4-Dichlorophenyl)-3-(2-methyl-1*H*-indol-1-yl)-1-(1*H*-1,2,4-triazol-1-yl)propan-2-ol (**11g**). White powder (15% yield); Rf = 0.30 (EtOAc/hexane: 1/1); mp: 170–171 °C; IR (KBr): ν 3422 (w, OH), 1582, 1271 cm^−1^; ^1^H NMR (DMSO-*d_6_*): δ 8.40 (s, 1H, H_3‴_), 7.82 (s, 1H, H_5‴_), 7.70 (d, 1H, *J* = 2.1 Hz, H_3′_), 7.49 (d, 1H, *J* = 7.2 Hz, H_6′_), 7.42 (d, 1H, *J* = 8.3 Hz, H_4″_), 7.31 (d, 1H, *J* = 7.2 Hz, H_5′_), 7.23 (dd, 1H, *J* = 8.3 Hz, *J* = 2.1 Hz, H_7″_), 6.95–7.03 (m, 2H, H_5″_, H_6″_), 6.24 (s, 1H, H_3″_), 6.06 (s, 1H, OH), 5.36 (d, 1H, *J* = 14.3 Hz, H_3_), 4.88 (d, 1H, *J* = 15.4 Hz, H_1_), 4.71 (d, 1H, *J* = 14.3 Hz, H_3_), 4.55 (d, 1H, *J* = 15.4 Hz, H_1_), 2.25 (s, 3H, CH_3_); ^13^C NMR (DMSO-*d_6_*): δ 12.9, 48.2, 53.5, 76.6, 100.7, 110.2, 118.9, 119.0, 120.0, 127.2, 127.8, 130.1, 131.1, 131.6, 133.3, 137.9, 138.2 (2C), 145.9, 150.8; MS *m*/*z*: 400, 402, 404 (M^+^), 255, 144 (100), 82. Anal. Calcd. for C_20_H_18_Cl_2_N_4_O (401.29): C: 59.86; H: 4.49; N: 13.95; found: C: 59.84; H: 4.44; N: 13.91%.

2-(2,4-Difluorophenyl)-3-(3-methyl-1*H*-indol-1-yl)-1-(1*H*-1,2,4-triazol-1-yl)propan-2-ol (**11h**). White powder (35% yield); Rf = 0.12 (EtOAc/hexane: 1/10); mp: 112–113 °C; IR (KBr): ν 3421 (w, OH), 1617, 1272 cm^−1^; ^1^H NMR (DMSO-*d_6_*): δ 8.33 (s, 1H, H_3‴_), 7.80 (s, 1H, H_5‴_), 7.18–7.30 (m, 2H, H_3′_, H_5′_), 7.44 (d, 1H, *J* = 7.3 Hz, H_4″_), 7.32 (d, 1H, *J* = 7.3 Hz, H_7″_), 7.12 (dd, 1H, *J* = 7.3 Hz, H_6″_), 7.07 (s, 1H, H_2″_), 6.99 (dd, 1H, *J* = 7.3 Hz, H_5″_), 6.85 (td, 1H, *J* = 7.9 Hz, *J* = 2.3 Hz, H_6′_), 6.25 (s, 1H, OH), 4.89 (d, 1H, *J* = 14.3 Hz, H_3_), 4.54 (s, 2H, H_1_), 4.51 (d, 1H, *J* = 14.3 Hz, H_3_), 2.23 (s, 3H, CH_3_); ^13^C NMR (DMSO-*d_6_*): δ 9.4, 52.0, 54.7, 75.2, 103.9 (dd, *^2^JCF = ^2^JCF =* 25.8 Hz), 109.2, 109.5, 110.9 (d, *^2^JCF =* 20.5 Hz), 118.2, 118.3, 121.0, 124.3 (dd, *^2^JCF =* 12.9 Hz, *^4^JCF =* 3.3 Hz), 127.3, 127.9, 130.1 (dd, *^3^JCF = ^3^JCF =* 6.2 Hz), 137.2, 145.0, 150.6, 159.1 (dd, *^1^JCF =* 246.5 Hz, *^3^JCF =* 12.4 Hz), 160.5 (dd, *^1^JCF =* 246.5 Hz, *^3^JCF =* 12.9 Hz); MS *m*/*z*: 368 (M^+^), 255, 144 (100), 82. Anal. Calcd. for C_20_H_18_F_2_N_4_O (368.38): C: 65.20; H: 4.89; N: 15.20; found: C: 65.18; H: 4.91; N: 15.22%.

2-(2,4-Dichlorophenyl)-3-(3-methyl-1*H*-indol-1-yl)-1-(1*H*-1,2,4-triazol-1-yl)propan-2-ol (**11i**). Red powder (24% yield); Rf = 0.65 (EtOAc/hexane: 1/10); mp: 161–162 °C; IR (KBr): ν 3399 (w, OH), 1586, 1275 cm^−1^; ^1^H NMR (DMSO-*d_6_*): δ 8.31 (s, 1H, H_3‴_), 7.77 (s, 1H, H_5‴_), 7.62 (d, 1H, *J* = 2.1 Hz, H_3′_), 7.46 (d, 1H, *J* = 7.5 Hz, H_7″_), 7.44 (d, 1H, *J* = 7.5 Hz, H_4″_), 7.42 (d, 1H, *J* = 8.9 Hz, H_6′_), 7.25 (dd, 1H, *J* = 8.9 Hz, *J* = 2.1 Hz, H_5′_), 7.13 (dd, 1H, *J = J =* 7.5 Hz, H_5″_), 7.04 (s, 1H, H_2″_), 7.01 (dd, 1H, *J = J =* 7.5 Hz, H_5″_), 6.33 (s, 1H, OH), 5.31 (d, 1H, *J* = 14.3 Hz, H_3_), 4.73 (s, 2H, H_1_), 4.50 (d, 1H, *J* = 14.3 Hz, H_3_), 2.22 (s, 3H, CH_3_); ^13^C NMR (DMSO-*d_6_*): δ 9.4, 50.7, 53.3, 76.7, 109.4, 109.9, 118.3 (2C), 121.2, 127.0, 127.4, 128.0, 129.9, 131.2, 131.4, 133.1, 137.0, 137.4, 145.1, 150.6; MS *m*/*z*: 400, 402, 404 (M^+^), 255, 144 (100), 82. Anal. Calcd. for C_20_H_18_Cl_2_N_4_O (401.29): C: 59.86; H: 4.49; N: 13.95; found: C: 59.83; H: 4.52; N: 13.98%.

2-(2,4-Difluorophenyl)-3-(2,3-dimethyl-1*H*-indol-1-yl)-1-(1*H*-1,2,4-triazol-1-yl)propan-2-ol (**11j**). Red powder (22% yield); Rf = 0.16 (EtOAc/hexane: 1/10); mp: 102–103 °C; IR (KBr): ν 3384 (w, OH), 1614, 1272 cm^−1^; ^1^H NMR (DMSO-*d_6_*): δ 8.38 (s, 1H, H_3‴_), 7.79 (s, 1H, H_5‴_), 7.20–7.31 (m, 2H, H_3′_, H_5′_), 7.39 (d, 1H, *J* = 6.4 Hz, H_4″_), 7.36 (d, 1H, *J* = 6.4 Hz, H_7″_), 7.09 (dd, 1H, *J = J =* 6.4 Hz, H_6″_), 6.86–7.01 (m, 2H, H_6′_, H_5″_), 6.06 (s, 1H, OH), 4.96 (d, 1H, *J* = 14.5 Hz, H_3_), 4.60 (d, 1H, *J* = 14.5 Hz, H_1_), 4.55 (d, 1H, *J* = 14.5 Hz, H_3_), 4.43 (d, 1H, *J* = 15.6 Hz, H_1_), 2.18 (s, 6H); ^13^C NMR (DMSO-*d_6_*): 8.8, 10.2, 49.9, 54.8, 75.6, 104.1 (dd, *^2^JCF = ^2^JCF =* 25.7 Hz), 106.1, 109.7, 111.0 (dd, *^2^JCF =* 20.5 Hz, *^4^JCF =* 2.9 Hz), 117.3, 118.5, 120.2, 124.9 (dd, *^3^JCF =* 9.1 Hz, *^3^JCF =* 12.9 Hz), 128.3, 130.1 (dd, *^2^JCF =* 22.4 Hz, *^4^JCF =* 3.8 Hz), 133.8, 137.2, 145.2, 150.6, 159.3 (dd, *^1^JCF =* 254.6 Hz, *^3^JCF =* 11.4 Hz), 160.8 (dd, *^1^JCF =* 246.5 Hz, *^3^JCF =* 8.5 Hz); MS *m*/*z*: 382 (M^+^), 269, 158 (100), 82. Anal. Calcd. for C_21_H_20_F_2_N_4_O (382.41): C: 65.95; H: 5.23; N: 14.64; found: C: 65.98; H: 5.25; N: 14.62%.

2-(2,4-Dichlorophenyl)-3-(2,3-dimethyl-1*H*-indol-1-yl)-1-(1*H*-1,2,4-triazol-1-yl)propan-2-ol (**11k**). White powder (15% yield); Rf = 0.17 (EtOAc/hexane: 1/1); mp: 123–124 °C; IR (KBr): ν 3429 (w, OH), 1584, 1273 cm^−1^; ^1^H NMR (DMSO-*d_6_*): δ 8.39 (s, 1H, H_3‴_), 7.80 (s, 1H, H_5‴_), 7.57 (d, 1H, *J* = 1.8 Hz, H_3′_), 7.51 (d, 1H, *J* = 7.9 Hz, H_4″_), 7.40 (d, 1H, *J* = 8.3 Hz, H_6′_), 7.37 (d, 1H, *J* = 7.9 Hz, H_7″_), 7.27 (dd, 1H, *J* = 8.3 Hz, *J* = 1.8 Hz, H_5′_), 7.11 (dd, 1H, *J = J =* 7.9 Hz, H_6″_), 7.00 (dd, 1H, *J = J =* 7.9 Hz, H_5″_), 6.03 (s, 1H, OH), 5.39 (d, 1H, *J* = 14.3 Hz, H_3_), 4.90 (d, 1H, *J* = 15.3 Hz, H_1_), 4.66 (d, 1H, *J* = 14.3 Hz, H_3_), 4.51 (d, 1H, *J* = 15.3 Hz, H_1_), 2.21 (s, 6H); ^13^C NMR (DMSO-*d_6_*): 10.3, 13.9, 48.2, 53.4, 76.7, 106.1, 109.4, 117.3, 118.5, 120.1, 127.2, 127.6, 130.0, 131.1, 131.2, 133.2, 137.3, 137.8, 140.4, 146.2, 150.6; MS *m*/*z*: 414, 416, 418 (M^+^), 269, 158 (100), 82. Anal. Calcd. for C_21_H_20_Cl_2_N_4_O (415.32): C: 60.73; H: 4.82; N: 13.48; found: C: 60.69; H: 4.79; N: 13.52%.

### 3.4. Synthesis of Compounds ***11l*** and ***11m*** (Route 2)

#### 3.4.1. Procedure for the *N*-1 and *N*-2-alkylation of 1*H*-1,2,3-Triazole

1-(2,4-Dichlorophenyl)-2-(1*H*-1,2,3-triazol-1-yl)ethanone (**9g**) and 1-(2,4-dichlorophenyl)-2-(2*H*-1,2,3-triazol-2-yl)ethan-1-one (**9h**) were synthesized according to methods a and b.

Method a: To a solution of 2-chloro-2′,4′-dichloroacetophenone (2.50 g, 11.19 mmol) in acetonitrile (40 mL) 1*H*-1,2,3-triazole (1.55 g, 22.37 mmol) and K_2_CO_3_ (3.09 g, 22.37 mmol) were added. The reaction mixture was irradiated for 50 min in a microwave oven (Discover, CEM) and programmed to obtain a temperature of 85 °C with a maximum power output of 50 W. After cooling, the mixture was filtered and the volatile fraction was evaporated under reduced pressure. The residue was diluted with H_2_O (40 mL) and extracted with EtOAc (3 × 80 mL), then the organic layer was washed with brine (80 mL), dried over anhydrous sodium sulfate, and the volatile fraction was evaporated under reduced pressure. ^1^H NMR (DMSO-*d_6_*) of the residue gave a ratio 49/51 of compounds **9g/9h**. The residue was purified by silica gel column chromatography (dichloromethane).

1-(2,4-Dichlorophenyl)-2-(1*H*-1,2,3-triazol-1-yl)ethanone (**9g**). White powder (49% yield); Rf = 0.06 (dichloromethane); mp: 118–120 °C; IR (KBr): ν 1717 (s, C=O), 1585, 1215 cm^−1^; ^1^H NMR (DMSO-*d_6_*): δ 8.16 (s, 1H, H_5″_); 8.05 (d, 1H, *J* = 8.5 Hz, H_6′_); 7.88 (d, 1H, *J* = 2.1 Hz, H_3′_); 7.84 (s, 1H, H_4″_); 7.70 (dd, 2H, *J* = 8.5 Hz, *J* = 2.1 Hz, H_5′_); 6.11 (s, 2H, CH_2_); ^13^C NMR (DMSO-*d_6_*): 57.5, 126.4, 127.7, 130.6, 131.5, 132.1, 133.3, 133.4, 137.5, 192.9.

1-(2,4-Dichlorophenyl)-2-(2*H*-1,2,3-triazol-2-yl)ethan-1-one (**9h**). White powder (50%); Rf = 0.29 (dichloromethane); mp: 100–101 °C; IR (KBr): ν 1717 (s, C=O), 1580, 1204 cm^−1^; ^1^H NMR (DMSO-*d_6_*): δ 7.99 (d, 1H, *J* = 8.5 Hz, H_6′_); 7.91 (s, 2H, H_3″_, H_4″_); 7.84 (d, 1H, J = 2.1 Hz, H_3′_); 7.65 (dd, 1H, *J* = 8.5 Hz, *J* = 2.1 Hz, H_5′_); 6.14 (s, 2H, CH_2_); ^13^C NMR (DMSO-*d_6_*): 62.1, 127.6, 130.5, 131.3, 132.0, 133.6, 135.3 (2C), 137.3, 193.5.

Method b: To a solution of 2-chloro-2′,4′-dichloroacetophenone (2.50 g, 11.19 mmol) in acetonitrile (40 mL) 1*H*-1,2,3-triazole (1.55 g, 22.37 mmol) and K_2_CO_3_ (3.09 g, 22.37 mmol) were added. The reaction mixture was heated at 85 °C for 8 h. After cooling, the mixture was filtered and the volatile fraction was evaporated under reduced pressure. The residue was diluted with H_2_O (40 mL) and extracted with EtOAc (3 x 80 mL), then the organic layer was washed with brine (80 mL), dried over anhydrous sodium sulfate, and the volatile fraction was evaporated under reduced pressure. ^1^H NMR (DMSO-*d_6_*) of the residue gave a ratio 61/39 of compounds **9g/9h**.

#### 3.4.2. General Procedure for the Synthesis of Oxiranes **10g** and **10h**

A procedure [27] similar to that for obtaining **10a** was used to prepare compounds **10g** and **10h**.

2-(2,4-Dichlorophenyl)-3-(1*H*-1,2,3-triazol-1-yl)-1,2-epoxypropane (**10g**). Orange oil (99% yield); Rf = 0.65 (EtOH/dichloromethane: 1/10); IR (NaCl): ν 1583, 1276 cm^−1^; ^1^H NMR (DMSO-*d_6_*): δ 8.05 (s, 1H, H_5″_); 7.73 (s, 1H, H_4″_); 7.72 (d, 1H, *J* = 2.1 Hz, H_3′_); 7.37 (dd, 1H, *J* = 8.2 Hz, *J* = 2.1 Hz, H_5′_); 7.09 (d, 1H, *J* = 8.2 Hz, H_6′_); 5.12 (d, 1H, *J* = 14.7 Hz, H_3_); 4.77 (d, 1H, *J* = 14.7 Hz, H_3_); 3.20 (d, 1H, *J* = 4.9 Hz, H_1_); 3.00 (d, 1H, *J* = 4.9 Hz, H_1_); ^13^C NMR (DMSO-*d_6_*): 51.9, 52.9, 58.5, 125.8, 127.3, 128.7, 130.8, 133.1, 133.4, 133.5, 134.0.

2-(2,4-Dichlorophenyl)-3-(2*H*-1,2,3-triazol-2-yl)-1,2-epoxypropane (**10h**). Orange oil (86% yield); Rf = 0.74 (EtOH/dichloromethane: 1/10); IR (NaCl): ν 1584, 1256 cm^−1^; ^1^H NMR (DMSO-*d_6_*): δ 7.76 (s, 2H, H_3″_, H_4″_); 7.67 (d, 1H, *J* = 2.1 Hz, H_3′_); 7.33 (dd, 1H, *J* = 8.2 Hz, *J* = 2.1 Hz, H_5′_); 7.08 (d, 1H, *J* = 8.2 Hz, H_6′_); 5.15 (d, 1H, *J* = 14.7 Hz, H_3_); 4.78 (d, 1H, *J* = 14.7 Hz, H_3_); 3.25 (d, 1H, *J* = 4.6 Hz, H_1_); 2.98 (d, 1H, *J* = 4.6 Hz, H_1_); ^13^C NMR (DMSO-*d_6_*): 51.4, 58.0, 62.1, 127.2, 128.5, 130.9, 133.8 (2C), 134.8 (3C).

#### 3.4.3. General Procedure for the Sysnthesis of Triazole Derivatives **11l** and **11m**

A procedure similar to **11a** was used to prepare compounds **11l** and **11m**.

2-(2,4-Dichlorophenyl)-3-(1*H*-indol-1-yl)-1-(1*H*-1,2,3-triazol-1-yl)propan-2-ol (**11l**). White powder (52% yield); Rf = 0.11 (EtOAc/hexane: 1/1); mp: 210–211 °C; IR (KBr): ν 3166 (w, OH), 1585, 1214 cm^−1^; ^1^H NMR (DMSO-*d_6_*): δ 7.87 (s, 1H, H_5‴_), 7.64 (d, 1H, J = 2.1 Hz, H_3′_), 7.58 (s, 1H, H_4‴_), 7.53 (d, 1H, *J* = 7.0 Hz, H_4″_), 7.51 (d, 1H, *J* = 7.0 Hz, H_7″_), 7.36 (d, 1H, *J* = 8.9 Hz, H_6′_), 7.23 (d, 1H, *J* = 2.8 Hz, H_2″_), 7.21 (dd, 1H, *J* = 8.9 Hz, *J* = 2.1 Hz, H_5′_), 7.14 (dd, 1H, *J = J =* 7.0 Hz, H_6″_), 7.01 (dd, 1H, *J = J =* 7.0 Hz, H_5″_), 6.47 (s, 1H, OH), 6.41 (d, 1H, *J* = 2.8 Hz, H_3″_), 5.61 (d, 1H, *J* = 13.7 Hz, H_3_), 4.90 (d, 1H, *J* = 14.4 Hz, H_1_), 4.83 (d, 1H, *J* = 14.4 Hz, H_1_), 4.65 (d, 1H, *J* = 13.7 Hz, H_3_); ^13^C NMR (DMSO-*d_6_*): 50.8, 53.7, 76.8, 101.2, 110.1, 118.9, 120.1, 121.1, 125.8, 126.9, 127.6, 129.6, 129.9, 131.2 (2C), 132.6, 133.1, 136.4, 137.0; MS *m*/*z*: 386, 388, 390 (M^+^), 241, 130 (100), 82. Anal. Calcd. for C_19_H_16_Cl_2_N_4_O (387.26): C: 58.92; H: 4.13; N: 14.46; found: C: 58.88; H: 4.10; N: 14.43%.

2-(2,4-Dichlorophenyl)-3-(1*H*-indol-1-yl)-1-(2*H*-1,2,3-triazol-2-yl)propan-2-ol (**11m**). White powder (56% yield); Rf = 0.59 (EtOAc/hexane: 1/1); mp: 159–161 °C; IR (KBr): ν 3434 (w, OH), 1589, 1215 cm^−1^; ^1^H NMR (DMSO-*d_6_*): δ 7.67 (s, 2H, H_3‴_, H_4‴_), 7.61 (d, 1H, J = 2.1 Hz, H_3′_), 7.53 (d, 1H, *J* = 7.6 Hz, H_4″_), 7.48 (d, 1H, *J* = 7.6 Hz, H_7″_), 7.32 (d, 1H, *J* = 8.5 Hz, H_6′_), 7.20 (d, 1H, *J* = 3.1 Hz, H_2″_), 7.17 (dd, 1H, *J* = 8.5 Hz, *J* = 2.1 Hz, H_5′_), 7.09 (dd, 1H, *J = J =* 7.6 Hz, H_6″_), 6.98 (dd, 1H, *J = J =* 7.6 Hz, H_5″_), 6.36 (d, 1H, *J* = 3.1 Hz, H_3″_), 6.28 (s, 1H, OH), 5.56 (d, 1H, *J* = 14.0 Hz, H_3_), 4.99 (d, 1H, *J* = 15.0 Hz, H_1_), 4.83 (d, 1H, *J* = 14.0 Hz, H_3_), 4.72 (d, 1H, *J* = 15.0 Hz, H_1_); ^13^C NMR (DMSO-*d_6_*): 50.8, 58.4, 77.1, 101.0, 110.2, 118.8, 120.1, 120.9, 126.8, 127.5, 129.5, 129.7, 131.3, 131.5, 132.9, 134.2 (2C); 137.0 (2C); MS *m*/*z*: 386, 388, 390 (M^+^), 241, 130 (100), 82. Anal. Calcd. for C_19_H_16_Cl_2_N_4_O (387.26): C: 58.92; H: 4.13; N: 14.46; found: C: 58.90; H: 4.15; N: 14.41%.

### 3.5. Chiral HPLC Chromatography

The chromatography system consisted of SpectraSystem (Thermo Electron S.A., Waltham, MA, USA) P1000-010XR2 isocratic pump and a dual wavelength SpectraSystem UV2000 detector. T data acquisition system was performed with an IBM PC/computer using Azur 3.0 (Datalys, Saint-Martin d’Héres, France) as chromatography sofware. Chromatography was performed on the Chiralcel OD-H column (250 × 4.6 mm, Daicel Chemical Industries Ltd, Tokyo, Japan) packed with 5 µm silica gel coated by cellulose tris(3,5-dimethylphenylcarbamate). A Rheodyne 9125 injector with a 20 μL sample loop was used. The mobile phases used were: (A) Acetonitrile, 100; (B) acetonitrile/diethylamine, 100:0.1 (*v*/*v*); (C) ethanol/diethylamine, 100:0.1 (*v*/*v*); (D) methanol/diethylamine, 100:0.1 (*v*/*v*); (E) n-hexane/ethanol/diethylamine, 60:40:0.1 (*v*/*v*/*v*); (F) n-hexane/methanol/ethanol/diethylamine, 75:15:10:0.1 (*v*/*v*/*v*/*v*). Solvents were of HPLC quality (Carlo Erba, Val de Reuil, France). The flow rate was 1.1 mL/min and the injection volume was 20 μL. The detection wavelength was 250 nm. The column temperature was at 25–30 °C. The sample concentration was 10 μg/mL in mobile phase. This method was used to separate the enantiomers of compound **8g**.

### 3.6. X-ray Crystallography Studies

The structure of compounds (+)-**8g** and (−)-**8g** was established by X-ray crystallography (Figure 2 and Figure 3) [44]. A colorless single crystal (0.25 × 0.10 × 0.05 mm^3^) of (+)-**8g** was obtained by slow evaporation from methanol/chloroform (30:70, *v*/*v*) solution: Monoclinic, space group P21, *a* = 6.707(2) Å, *b* = 32.174(4) Å, *c* = 10.080(2) Å, α = 90.0°, β = 109.40(2)°, γ = 90.0°, *V* = 2051.7(8) Å^3^, Z = 2, δ(calcd) = 1.254 Mg.m^-3^, FW = 774.52 for C_38_H_32_Cl_4_N_8_O_2_, *F*(000) = 800. A colorless single crystal (0.25 × 0.10 × 0.07 mm^3^) of (−)-**8g** was obtained by slow evaporation from methanol/chloroform (30:70, *v/v*) solution: Monoclinic, space group P21, *a* = 6.681(3) Å, *b* = 32.142(4) Å, *c* = 10.111(4) Å, α = 90.0°, β = 109.40(2)°, γ = 90.0°, *V* = 2048.0(13) Å^3^, Z = 2, δ(calcd) = 1.256 Mg.m^−3^, FW = 774.52 for C_38_H_32_Cl_4_N_8_O_2_, *F*(000) = 800. The unit cell dimensions were determined using the least-squares fit from 25 reflections (25° < θ < 35°). Intensities were collected with an Enraf–Nonius CAD-4 diffractometer using the CuKα radiation and a graphite monochromator up to θ = 55°. No intensity variation of 2 standard reflections monitored every 90 min was observed. The data were corrected for Lorentz and polarization effects and for empirical absorption correction [45]. The structure was solved by direct methods Shelx 86 [46] and refined using Shelx 93 [47] suite of programs.

### 3.7. Biological Assays

#### 3.7.1. Anti-*Candida* In Vitro Assay

Test fungal strains were obtained from the American Type Culture Collection (ATCC) or were clinical isolates from the Laboratory of Parasitology and Medical Mycology, Centre Hospitalier Universitaire (CHU) of Nantes. The strains were maintained on Sabouraud agar slants and were subcultured 24 h before used. The activity of the target compounds **8a**–**g** and **11a**–**m** was determined by the method previously described [48]. Briefly, molecules were diluted in RPMI 1640 medium supplemented with 0.165 M morpholinopropanesulphonic acid (Sigma-Aldrich, Saint-Quentin Fallavier, France), 2% glucose, and antibiotics. *Candida* suspensions were prepared in RPMI 1640 medium (Sigma-Aldrich, Saint-Quentin Fallavier, France) adjusted to give a final concentration of 10^3^ cells/mL, and a 96-well microplate (Nunc, D. Dutscher, Brumath, France) was seeded with 100 µL. Each concentration of molecules (100 µL) to be tested was added (in triplicate) and plates were incubated at 37 °C for 24 h. The cellular viability was evaluated on the Fluorolite 1000 (Dynatech, France) with an excitation at 550 nm and an emission at 590 nm after a 4 h incubation with 10 µL of Alamar Blue^®^. The minimal inhibitory concentration (MIC) is the concentration that inhibited 80% of the cell growth and was determined by linear regression analysis. MIC was expressed as the mean of the triplicate values. KTC and FLC were used as standards.

#### 3.7.2. MRC5 Toxicity Assay

The cytotoxicity of compounds was studied with human fibroblast (MRC5). Cells were grown in RPMI 1640 medium (Sigma-Aldrich, Saint-Quentin Fallavier, France) supplemented with 10% fetal bovin serum (Sigma–Aldrich). Drugs were tested at three concentrations (100, 10, and 1 µM) in triplicate. After a 96-h incubation time, cytotoxicity was measured on the Fluorolite 1000 (Dynatech, Guyancourt, France) after a 4-h incubation time with Uptiblue^®^ (Interchim, Montluçon, France). Inhibitory concentration 50 is a mean of triplicate values. A toxicity index was determined as follow: IC_50_ against MRC5/Geometric mean of the MICs against *Candida* spp. [28].

#### 3.7.3. In Vitro Inhibition of Aromatase

The assay was performed according to previously described methods [49,50,51] by monitoring the enzyme activity by measuring the ^3^H_2_O formed from [1β-^3^H]androstenedione during aromatization. In brief, the reaction mixture, containing [1β-^3^H]androstenedione (0.08 µCi, 15 nM), unlabeled androstenedione (485 nM), the NADPH-generating system (2 mM), the inhibitor (0–100 µM), and phosphate buffer (0.05 M, pH 7.4), was preincubated for 5 min at 30 °C. Microsomal protein (0.1 mg) was added to start the reaction. After incubation of 15 min at 30 °C, the reaction was stopped by adding 200 µL of a cold HgCl_2_ solution (1 mM). After the addition of 200 µL of an aqueous dextran-coated charcoal (DCC) suspension (2%), the vials were shaken then centrifuged to separate the charcoal-absorbed steroids. Aliquots of the supernatant were assayed for ^3^H_2_O by counting in a scintillation mixture using a LKB-Wallac β-counter.

#### 3.7.4. In Vitro Inhibition of 17α-Hydroxylase/17,20-Lyase

The assay was performed similar to described methods [29,52]. In summary, the reaction mixture containing progesterone (1.25 mM), NADPH (125 nmol), the inhibitor, and phosphate buffer (pH 7.4) was preincubated for 5 min at 32 °C. Microsomal protein was added to start the reaction. After incubation for 20 min at 32 °C, the reaction was stopped by adding 50 µL of 1 M aqueous HCl solution. After the addition of 1 mL EtOAc, the vials were shaken then centrifuged. The organic phase was removed, vortexed with phosphate buffer (250 µL), 1 M aqueous HCl (50 µL), and then dried. Aliquots of 25 µL methanol, containing 250 pmol of fluorocortisol acetate as internal standard, were added to the extracts. The samples (20 µL) were submitted to HPLC (RP-8 column, methanol/water 1:1, *v*/*v*) and measured using UV (240 nm). For compounds causing an inhibition over 80%, IC_50_ values were determined.

#### 3.7.5. MCF-7 Cell Culture and In Vitro Assay for Inhibition of Metabolism of All-*trans* Retinoic Acid (ATRA)

Human MCF-7 breast cancer cells were cultured in phenol red-free RPMI 1640 medium supplemented with 5% (*v*/*v*) charcoal free fetal calf serum, antibiotics (penicillin and streptomycin), and fungizone at the same concentration of 10 iU/mL (all reagents from Gibco Europe Ltd., Scotland, UK). Cells were grown in a humidified incubator (5% CO_2_, 95% air) at 37 °C.

[11,12-^3^H]All-*trans*-retinoic acid (37 MBq/mL) was purchased from PerkinElmer Life Science Ltd. (USA). All-*trans* retinoic acid, NADPH, butylated hydroxyanisole, and KTC were obtained from Sigma–Aldrich (Gillingham, UK). Liarozole was a gift from Stiefel Laboratories, High Wycombe, UK. MCF-7 cells were seeded in 12-well cell culture plates (Cornings Inc., New York, USA) at 2.5 × 10^5^ cells per well in a total volume of 1.5 mL. Cells were allowed to adhere to the well for 24 h. After 24 h, the medium from each well was removed, washed once with phosphate buffer saline (PBS), and replaced by fresh medium plus 10 μL inhibitor/solvent (acetonitrile) and 10 μL of ATRA (to give a final concentration of 1 × 10^−7^ M ATRA and 0.1 μCi [11,12-^3^H]all-*trans*-retinoic acid). The plates were foil wrapped and incubated at 37 °C for 9 h. Each treatment was performed in duplicate. The incubation was stopped by addition of 1 % acetic acid (100 μL/well), the medium was removed into separate glass tubes. Distilled water (200 μL) was added to each well and the cells scrapped off and the contents added to the appropriate glass tube. This procedure was repeated with a further 400 μL water but without scraping. Ethyl acetate containing 0.05 % (*w*/*v*) butylated hydroxyanisole (2 × 2 mL) was added to each tube. After vortexing for 15 s, the tubes were spun down at 3000 rpm for 15 min. The organic layer was then evaporated using a Christ centrifuge connected to a vacuum pump and a multitrap at −80 °C.

The HPLC system was equipped with a high-pressure pump (Milton–Roy pump, Stone, UK), injector with a 50 μL loop connected to a beta-RAM radioactivity detector, connected to a Compaq^™^ computer running Laura^®^ data acquisition and analysis software. This enabled online detection and quantification of radioactive peaks. The 10 μM C_18_ μBondapak^™^ 3.9 × 300 mm HPLC column (Waters, UK) was used for the all-*trans* retinoic acid metabolism studies. The HPLC column operating at an ambient temperature was used to separate the metabolites, which were eluted with acetonitrile/1% ammonium acetate in water/acetic acid (75:25:0.1, *v*/*v*/*v*) at a flow rate of 1.9 mL/min. The Ecoscint™ was used as the flow scintillation fluid.

The separated [^3^H]-metabolites were quantitatively calculated from the areas under the curves. Using a control with acetonitrile instead of inhibitor, these results were expressed as the “percentage inhibition relative to control” = 100[(metabolites (control)—metabolites (inhibitor)/(metabolites control)]%. KTC and liarozole were used as standards [53].

#### 3.7.6. In Vitro Inhibition of CYP11B1 and CYP11B2

For the CYP11B1 assay, 11-deoxycortisol (RSS) was used as substrate, whereas 11-deoxycorticosterone (DOC) was used for the CYP11B2 assay. These non-radioactive steroids were purchased from Sima (Deisenhofen, Germany). Radioactive steroids were obtained from Amersham Pharmacia Biotech (Freiburg, Germany). Two *Schizosaccharomyces pombe* strains were used, SZ1 expressing CYP11B1 and MB164 expressing CYP11B2. The assays were performed as described previously by Bureik et al. [54]. Exponentially growing fission yeast cells were cultivated by shaking and with good aeration at 30 °C in 500 µL Edinburgh Minimal Medium Glutamate (EMMG) containing supplements as required. Inhibitors were dissolved in ethanol at different concentrations, and equal volumes of ethanolic solutions were used in all cases (including controls). Final concentrations of inhibitors ranged from 100 nM to 5 µM. Cells were preincubated with the respective inhibitor solutions for 15 min prior to the addition of steroid substrates (100 nM DOC or 100 nM RSS, respectively). For detection of steroids, 1% of the total substrate amount was radioactively labeled as [^3^H]RSS, [^3^H]DOC, or [^14^C]DOC. After 4 h (MB164) or 24 h (SZ1) cultures were extracted with chloroform and dried under vacuum. The residues were dissolved in 10 µL chloroform and spotted onto glass-backed silica-coated HPTLC plates (Kieselgel 60 F_254_, Merck; Darmstadt, Germany). In addition, small amounts of non-radioactive steroids were spotted as references. The HPTLC was developed twice in chloroform/methanol/water (300:20:1, *v*/*v*/*v*) and steroids identified after exposure to Fuji imaging plates and quantitated on a phosphoimager (BAS-2500, Fuji; Stamford, CT, USA).

### 3.8. Computational Methods

#### 3.8.1. Molecular Docking

First molecular docking was performed for the compound (*R*)-**8g** and (*S*)-**8g** to assess their binding feasibility and difference. A six-step procedure was applied. (1) Three crystal structures of *C. albicans* CYP51 (PDB code: 5TZ1, 5FSA, 5V5Z) were taken from Pocketome [55], in which different PDB structures of the same protein were superimposed by the binding sites to take the induce-fit effect into consideration. (2) All structures were combined to calculate grid potential ensembles for the docking of (*R*)-**8g** and (*S*)-**8g**. The grid maps were calculated on a 0.5 Å 3D grid, containing: (i) van der Waals potentials; (ii) electrostatic potentials; (iii) hydrogen bonding potentials; and (iv) hydrophobic potential grids. (3) The structures of ligands were taken from crystallography [43]. (4) Four distinctive starting poses were generated for sampling. (5) The ligand was then sampled in the pre-generated grid potential ensembles through biased probability Monte Carlo sampling method [56] to optimize the positional variables of the ligand. (6) After the sampling, the ligand conformation was re-scored with the full-atom ICM scoring function [57]. All docking and scoring were performed in ICM-Pro v3.8-7c.

#### 3.8.2. Molecular Dynamics Simulation

Molecular dynamics simulation was performed as previously described [21]. Briefly, docking studies were performed using MOE [40] and CaCYP51 (PDB 5FSA [39]) to generate pdb files of the CaCYP51 crystal structure complexed with (*R*)-**8g** and (*S*)-**8g**. Molecular dynamics simulations were run on the CaCYP51-ligand complexes with the pdb files first optimized with protein preparation wizard in Maestro by assigning bond orders, adding hydrogen, and correcting incorrect bond types. A default quick relaxation protocol was used to minimise the MD systems with the Desmond program [41]. Force-field parameters for the complexes were assigned using the OPLS_ 2005 forcefield, that is, a 100 ns molecular dynamic run in the NPT ensemble (T = 300 K) at a constant pressure of 1 bar. Energy and trajectory atomic coordinate data were recorded at each 1.2 ns.

## 4. Conclusions

A series of 2-aryl-3-azolyl-1-indolyl-propan-2-ols was designed as new analogs of FLC by replacing one of its two triazole moieties by an indole scaffold. A first chemical approach was developed in seven steps, involving the synthesis of the key intermediate 1-(1*H*-benzotriazol-1-yl)methyl-1*H*-indole **4** and the final opening of oxiranes **7** by imidazole or 1*H*-1,2,4-triazole. A shorter process was also developed to access the target compounds in only three steps, this time with the ring opening by indole and analogs. Twenty azole derivatives were synthesized and tested against *C. albicans* and other *Candida* species. Thirteen compounds demonstrated a high level of activity against *C. albicans* CA98001, with MIC < 0.027 µg/mL, by comparison with the MIC value of FLC (MIC = 0.020 µg/mL). The in vitro cytotoxicity of compounds **8a**–**g** and **11a**–**k** was further evaluated on MRC-5 cells.

The enantiomers of the best anti-*Candida* compound, 2-(2,4-dichlorophenyl)-3-(1*H*-indol-1-yl)-1-(1*H*-1,2,4-triazol-1-yl)-propan-2-ol **8g**, were analyzed by X-ray diffraction to determine their absolute configuration. The (−)-**8g** enantiomer (MIC = 0.000256 µg/mL against *C. albicans* CA98001) was found with the *S*-absolute configuration. Additionally, molecular docking and MD simulation were performed and confirmed that the (*S*)-enantiomer aligned with the positioning of posaconazole within both the heme and access channel binding sites. These in silico results are consistent with the biological results presented. The selectivity of compounds **11d**, **8g**, and its enantiomers was also investigated against five human P450-dependent enzymes (CYP19, CYP17, CYP26A1, CYP11B1, and CYP11B2).

Overall, (*S*)-**8g** had a pharmacological profile to pursue further biological investigations such as a murine candidiasis assay and cytochrome P450 inhibition assays (e.g. CYP3A4, CYP2D6). Recently, we published extended biological exploration of **8g** (in vitro and in vivo assays) and then we confirmed the full potential of this molecule [28]. Another major challenge is the emergence of resistance to azole antifungals among *Candida* species [58]. Then a multi-disciplinary approach with joint expertise and networks is crucial to assist efficiently medicinal chemists for new optimizations. According to the 3D structures of CaCYP51 and our results obtained (Figure 9), further pharmacomodulation works should be performed to explore both the position of the azolyl chain on indole scaffold and the introduction of a long chain to occupy the space of the access channel (as does the N-arylpiperazine chain of posaconazole). In this regard, NMR will be of special interest for studying ligand access channels in cytochrome P450 enzymes [59,60,61,62].

A further biological investigation could be performed by testing a selection of our best 2-aryl-3-azolyl-1-indolyl-propan-2-ols (e.g. **8d**, **8f**, (−)-(*S*)-**8g**, **11g**, **11h**, **11i**) against the emerging *Candida auris* strain.

The last point is that the antifungal azoles targeting CaCYP51 could be a starting point for developing new treatments in some particular diseases such as human infections with protozoa (Trypanosomatidae) [63] and primary amoebic meningoencephalitis (PAM) [64]. The needs for new treatments are enormous in the field of parasitic diseases, and CYP51 is a promising target for further drug development.

## Figures and Tables

**Table 1:** Anti-*Candida* activity of propanol derivatives **8a**–**g** and **11a**–**m**.

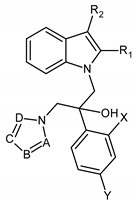									
Compound	A	B	C	D	R_1_	R_2_	X	Y	MIC ^1^ (µg/mL) *C. albicans* CA98001
**8a**	CH	CH	N	CH	H	H	H	F	0.03
**8b**	CH	CH	N	CH	H	H	H	Cl	0.02
**8c**	CH	CH	N	CH	H	H	H	Br	0.02
**8d**	CH	CH	N	CH	H	H	F	F	0.00035
**8e**	CH	CH	N	CH	H	H	Cl	Cl	0.062
**11e**	CH	CH	N	CH	H	H	H	CF_3_	0.23
**11a**	N	CH	N	CH	H	H	H	F	0.21
**11b**	N	CH	N	CH	H	H	H	Cl	0.024
**11c**	N	CH	N	CH	H	H	H	Br	0.027
**11d**	N	CH	N	CH	H	H	F	F	0.0198
**8g**	N	CH	N	CH	H	H	Cl	Cl	0.000259
**(+)-(*R*)-8g**	N	CH	N	CH	H	H	Cl	Cl	0.023
**(−)-(*S*)-8g**	N	CH	N	CH	H	H	Cl	Cl	0.000256
**8f**	N	CH	N	CH	H	H	H	CF_3_	0.009
**11f**	N	CH	N	CH	CH_3_	H	F	F	0.022
**11g**	N	CH	N	CH	CH_3_	H	Cl	Cl	0.0058
**11h**	N	CH	N	CH	H	CH_3_	F	Cl	0.0011
**11i**	N	CH	N	CH	H	CH_3_	Cl	Cl	0.007
**11j**	N	CH	N	CH	CH_3_	CH_3_	F	F	0.157
**11k**	N	CH	N	CH	CH_3_	CH_3_	Cl	Cl	1.246
**11l**	N	N	CH	CH	H	H	Cl	Cl	>100
**11m**	N	CH	CH	N	H	H	Cl	Cl	>100
**KTC**									0.005
**FLC**									0.02

^1^ Minimum inhibitory concentration (MIC = IC_80_, µg/mL) was expressed as the mean of triplicate measures.

**Table 2:** MIC ^1^ (µg/mL) spectrum of compounds **8a**–**g**, **11a**–**k**, ketoconazole (KTC), and FLC.

Compound	ATCC2091	CA98002	CA98005	ATCC6258	CK98002	CK98003	CL98001	ATCC90018
**8a**	0.131	0.205	0.148	>33.5	12.761	28.597	0.231	2.11
**8b**	0.215	0.165	0.106	>35.2	2.723	>35.183	0.222	1.3
**8c**	0.357	0.25	0.21	>39.6	5.076	36.565	0.25	1.84
**8d**	0.726	0.609	0.439	3.863	5.692	5.205	0.068	0.614
**8e**	0.19	0.251	0.274	1.2	1.8	0.28	0.116	0.24
**11e**	2.12	0.32	0.258	22.121	18.306	16.225	0.173	2.466
**11a**	0.437	0.404	0.437	19.24	21.426	18.668	0.259	2.523
**11b**	0.258	0.261	0.222	2.434	12.666	2.434	0.208	2.046
**11c**	0.477	0.338	0.286	1.51	2.463	2.503	0.215	2.026
**11d**	0.021	0.026	0.064	2.181	1.55	24.05	0.021	0.201
**8g**	<0.004	<0.004	<0.004	0.08	2.788	3.563	<0.004	0.15
**(+)-(*R*)-8g**	1.007	2.75	2.707	24.398	22.074	19.363	9.682	>38.726
**(−)-(*S*)-8g**	<0.039	<0.039	0.026	0.167	0.022	0.039	0.024	0.027
**8f**	0.247	0.233	0.749	19.091	10.557	3.068	0.199	2
**11f**	0.199	0.302	0.516	17.572	7.589	5.452	0.232	2.505
**11g**	0.526	0.321	0.269	>40	>40	>40	0.181	>40
**11h**	0.136	0.033	0.265	1.4	0.81	0.7	0.023	0.309
**11i**	0.026	0.044	0.03	3.01	0.803	1.685	0.025	0.245
**11j**	0.256	0.382	2.409	17.97	11.472	13.92	0.256	4.015
**11k**	2.741	1.121	2.99	25.293	20.226	>41.532	2.866	9.76
**KTC**	0.008	0.009	0.006	0.079	0.37	0.43	0.007	0.064
**FLC**	0.07	1	0.57	7.5	2	6.7	0.55	0.6

^1^ MIC (IC_80_, µg/mL) was expressed as the mean of triplicate measures. ATCC strains are reference strains (American Type Culture Collection). ATCC2091 is a *Candida albicans* strain. ATCC6258 is a *Candida krusei* strain. ATCC90018 is a *Candida parapsilosis* strain. CA = *Candida albicans*. CK = *Candida krusei*. CL = *Candida lusitaniae*.

**Table 3:** Cytotoxicity activity on MRC-5 cells of propanol derivatives **8a**–**g** and **11a**–**k**.

Compound	IC_50_^1^ (µM)
**8a**	50
**8b**	37
**8c**	31
**8d**	34
**8e**	31
**11e**	36
**11a**	>100
**11b**	>100
**11c**	38
**11d**	197.2
**8g**	35.0 ^2^
**(+)-(*R*)-8g**	32
**(−)-(*S*)-8g**	30
**8f**	76
**11f**	190.7
**11g**	157
**11h**	105.4
**11i**	19
**11j**	25
**11k**	97.3
**KTC**	69.1
**FLC**	>100

^1^ IC_50_ (half-maximal inhibitory concentration) values (µM) were expressed as the mean of triplicate measures. ^2^ Cytotoxicity activity of **8g** previously determined [28].

**Table 4:** Selectivity of compounds **11d**, **8g**, and its enantiomers on a panel of human P450-dependent enzymes (CYP19, CYP17, CYP26A1, CYP11B1, and CYP11B2).

Compound	CYP19 ^1^ IC_50_ (µM) (% Inhibition)	CYP17 ^2^ IC_50_ (µM) (% Inhibition)	CYP26A1 ^3^ IC_50_ (µM)	CYP11B1 ^4^ IC_50_ (µM) (Inhibition Effect)	CYP11B2 ^4^ IC_50_ (µM) (Inhibtion Effect)
**11d**	3.58	-	-	-	-
	(89)	(no inhibition)			
**8g**	-	-	-	-	-
	(27)	(<10%)		no inhibition	slight inhibition
**(+)-(*R*)-8g**	-	-	34	-	-
	(51)	(<5% inhibition)	-	no inhibition	slight inhibition
**(−)-(*S*)-8g**	-	-	18	-	-
	(72)	(<5% inhibition)	-	no inhibition	no inhibition
**AG**	29.75	-	-	-	-
**fadrozole**	0.030	not active	-	-	-
**anastrozole**	0.163	-	-	-	-
**letrozole**	0.025	-	-	-	-
**liarozole**	-	-	7	-	-
**BW19**	-	0.15	-	-	-
**KTC**	-	4.5	10	-	-

^1^ Values are the mean of at least two experiments performed in duplicate. % inhibition was determined at 36 µM. ^2^ Values are the mean of two experiments performed in duplicate. % inhibition was determined at 2.5 µM. ^3^ The IC_50_ values are the mean (±5%) of two experiments. ^4^ The results were obtained from three to four independent experiments.
